# Multiple functional neurosteroid binding sites on GABA_A_ receptors

**DOI:** 10.1371/journal.pbio.3000157

**Published:** 2019-03-07

**Authors:** Zi-Wei Chen, John R. Bracamontes, Melissa M. Budelier, Allison L. Germann, Daniel J. Shin, Krishnan Kathiresan, Ming-Xing Qian, Brad Manion, Wayland W. L. Cheng, David E. Reichert, Gustav Akk, Douglas F. Covey, Alex S. Evers

**Affiliations:** 1 Department of Anesthesiology, Washington University in St Louis, St Louis, Missouri, United States of America; 2 Taylor Family Institute for Innovative Psychiatric Research, St Louis, Missouri, United States of America; 3 Department of Developmental Biology, Washington University in St Louis, St Louis, Missouri, United States of America; 4 Department of Radiology, Washington University in St Louis, St Louis, Missouri, United States of America; Stanford University, UNITED STATES

## Abstract

Neurosteroids are endogenous modulators of neuronal excitability and nervous system development and are being developed as anesthetic agents and treatments for psychiatric diseases. While gamma amino-butyric acid Type A (GABA_A_) receptors are the primary molecular targets of neurosteroid action, the structural details of neurosteroid binding to these proteins remain ill defined. We synthesized neurosteroid analogue photolabeling reagents in which the photolabeling groups were placed at three positions around the neurosteroid ring structure, enabling identification of binding sites and mapping of neurosteroid orientation within these sites. Using middle-down mass spectrometry (MS), we identified three clusters of photolabeled residues representing three distinct neurosteroid binding sites in the human α_1_β_3_ GABA_A_ receptor. Novel intrasubunit binding sites were identified within the transmembrane helical bundles of both the α_1_ (labeled residues α_1_-N^408^, Y^415^) and β_3_ (labeled residue β_3_-Y^442^) subunits, adjacent to the extracellular domains (ECDs). An intersubunit site (labeled residues β_3_-L^294^ and G^308^) in the interface between the β_3_(+) and α_1_(−) subunits of the GABA_A_ receptor pentamer was also identified. Computational docking studies of neurosteroid to the three sites predicted critical residues contributing to neurosteroid interaction with the GABA_A_ receptors. Electrophysiological studies of receptors with mutations based on these predictions (α_1_-V^227^W, N^408^A/Y^411^F, and Q^242^L) indicate that both the α_1_ intrasubunit and β_3_-α_1_ intersubunit sites are critical for neurosteroid action.

## Abstract 2

Novel neurosteroid analogue photolabeling reagents identify three specific neurosteroid binding sites on α1β3 GABAA receptors, showing that a site between the α and β subunits, as well as a site within the α-subunit, contribute to neurosteroid-mediated enhancement of GABAA currents.

## Author summary

Neurosteroids are cholesterol metabolites produced by neurons and glial cells that participate in central nervous system (CNS) development, regulate neuronal excitability, and modulate complex behaviors such as mood. Exogenously administered neurosteroid analogues are effective sedative hypnotics and are being developed as antidepressants and anticonvulsants. Gamma amino-butyric acid Type A (GABA_A_) receptors, the principal ionotropic inhibitory neurotransmitter receptors in the brain, are the primary functional target of neurosteroids. Understanding the molecular details of neurosteroid interactions with GABA_A_ receptors is critical to understanding their mechanism of action and developing specific and effective therapeutic agents. In the current study, we developed a suite of neurosteroid analogue affinity labeling reagents, which we used to identify three distinct binding sites on GABA_A_ receptors and to determine the orientation of neurosteroid binding in each site. Electrophysiological studies performed on receptors with mutations designed to disrupt the identified binding sites showed that two of the three sites contribute to neurosteroid modulation of GABA_A_ currents. The distinct patterns of neurosteroid affinity, binding orientation, and effect provide the potential for the development of isoform-specific agonists, partial agonists, and antagonists with targeted therapeutic effects.

## Introduction

Neurosteroids are cholesterol metabolites produced by neurons [1] and glia [2] in the central nervous system (CNS) that are thought to play important roles in both nervous system development and behavioral modulation [3]. Neurosteroid analogues are also being developed as sedative hypnotics [4], antidepressants [5], and anticonvulsants [6]. Gamma amino-butyric acid Type A (GABA_A_) receptors, the principal ionotropic inhibitory neurotransmitter receptors in the CNS, have been identified as the primary functional target of neurosteroids. The major endogenous neurosteroids—allopregnanolone and tetrahydroxy-desoxycorticosterone (THDOC)—are positive allosteric modulators (PAMs) of GABA_A_ receptors, potentiating the effects of GABA at nanomolar concentrations and directly activating currents at micromolar concentrations. GABA_A_ receptors are members of the pentameric ligand-gated ion channel (pLGIC) superfamily and are typically composed of two α subunits, two β subunits, and one γ or δ subunit [7]. There are 19 homologous GABA_A_ receptor subunits (including six α, three β and two γ isoforms), with each subunit composed of a large extracellular domain (ECD), a transmembrane domain (TMD) formed by four membrane-spanning helices (TMD1–4), a long intracellular loop between TMD3 and TMD4, and a short extracellular C-terminus. These distinctive structural domains form binding sites for a number of ligands: GABA and benzodiazepines bind to the ECD, picrotoxin to the channel pore [8], and general anesthetics—such as propofol [9, 10], etomidate [11], barbiturates [12], and neurosteroids—to the TMDs [13–18].

Substantial evidence indicates that neurosteroids produce their effects on GABA_A_ receptors by binding to sites within the TMDs [13–15, 19, 20]. Whereas the TMDs of β-subunits are critically important to the actions of propofol and etomidate [11, 21–26], the α-subunit TMDs appear to be essential for neurosteroid action. Mutagenesis studies in α_1_β_2_γ_2_ GABA_A_ receptors identified several residues in the α_1_ subunit, notably Q^241^ in TMD1, as critical to neurosteroid potentiation of GABA-elicited currents [14, 27]. More recent crystallographic studies have shown that, in homo-pentameric chimeric receptors in which the TMDs are derived from either α_1_ [16] or α_5_ subunits [17], the neurosteroids THDOC and pregnanolone bind in a cleft between the α-subunits, with the C3-hydroxyl substituent of the steroids interacting directly with α_1_^Q241^. Neurosteroids are PAMs of these chimeric receptors, and α_1_^Q241L^ and α_1_^Q241W^ mutations eliminate this modulation. These studies posit a single critical binding site for neurosteroids that is conserved across the six α-subunit isoforms [14, 27].

A significant body of evidence also suggests that neurosteroid modulation of GABA_A_ receptors may be mediated by multiple sites. Site-directed mutagenesis identified multiple residues that affect neurosteroid action on GABA_A_ receptors, suggestive of two neurosteroid binding sites, with one site mediating potentiation of GABA responses and the other mediating direct activation [14, 27]. Single channel electrophysiological studies as well as studies examining neurosteroid modulation of [^35^S]t-butylbicyclophosphorothionate (TBPS) binding, have also identified multiple distinct effects of neurosteroids with various structural analogues producing some or all of these effects, consistent with multiple neurosteroid binding sites [28–30]. Finally, neurosteroid photolabeling studies in the bacterial pLGIC, *Gloeobacter* ligand-gated ion channel (GLIC), demonstrate two neurosteroid binding sites per monomer [31], one analogous to the canonical intersubunit site and one located in an intrasubunit pocket previously shown to bind propofol [32, 33] and the inhalational anesthetics [33, 34]. Both of these sites contribute to neurosteroid modulation of GLIC currents, suggesting the possibility of analogous sites in GABA_A_ receptors.

We have developed a suite of neurosteroid analogue photolabeling reagents with photolabeling groups positioned around the neurosteroid ring structure to identify all of the neurosteroid binding sites on GABA_A_ receptors and determine the orientation of neurosteroid binding within each site. Photolabeling was performed in membranes from a mammalian cell line that stably expresses α_1His-FLAG_β_3_ receptors (rather than in detergent-solubilized receptors) to optimize the likelihood that the receptors were in native conformations and environment. Finally, we deployed a middle-down mass spectrometry (MS) approach, coupled with a stable-heavy isotope encoded click chemistry tag for neurosteroid-peptide adduct identification to circumvent challenges associated with MS identification (predominantly neutral loss) and quantification of neurosteroid-peptide adducts [35]. Using these approaches, we have identified three clusters of neurosteroid-photolabeled residues on the human α_1_β_3_ GABA_A_ receptor. Computational docking studies, guided by the photolabeling data, were used to describe three binding sites and the orientation of the neurosteroids within each site. The docking studies were also used to predict critical residues to test the contribution of each of these sites to neurosteroid modulation of GABA_A_ currents. Site-directed mutagenesis of these sites and electrophysiological studies indicate that at least two of three structurally distinct sites contribute to allosteric modulation of GABA currents.

## Results

### Development and characterization of allopregnanolone-analogue photolabeling reagents

**Fig 1:**
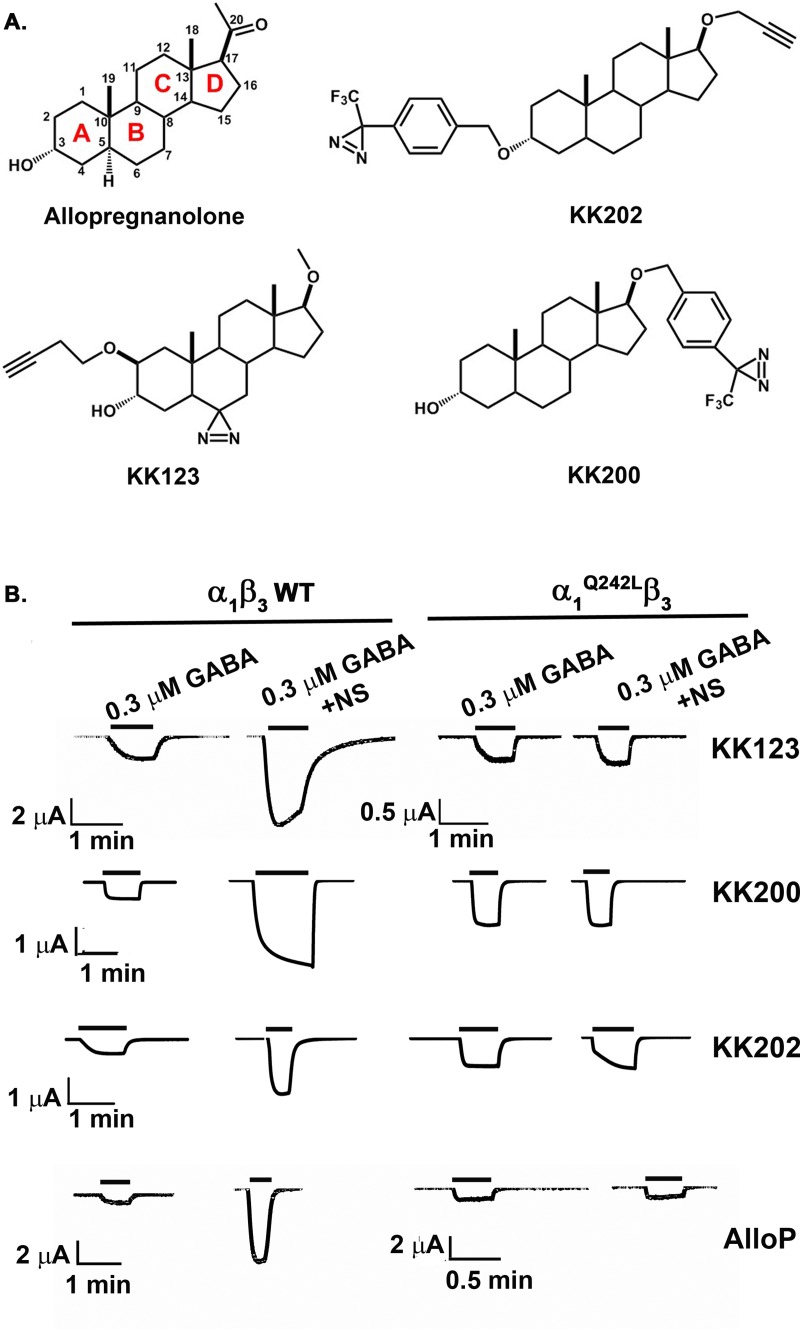
Allopregnanolone-analogue photolabeling reagents. (A) The structure of allopregnanolone, KK123, KK200, and KK202. (B). Allopregnanolone (1 μM) and the photolabeling reagents (10 μM) potentiate GABA-elicited currents of α_1_β_3_-GABA_A_ receptors; potentiation is blocked by mutation of α_1_^Q242L^β_3_, indicating that these photolabeling reagents mimic the action of allopregnanolone. The numerical data are included in S1 Data. AlloP, allopregnanolone; GABA_A_, gamma amino-butyric acid Type A; NS, neurosteroid; WT, wild-type.

Allopregnanolone (3α-hydroxy-5α-pregnan-20-one) is a potent, endogenous PAM of GABA_A_ receptors (Fig 1A). We synthesized three photolabeling analogues of allopregnanolone in which photolabeling moieties were placed at various positions around the steroid backbone. KK123 has a 6-diazirine photolabeling group on the C5-C6-C7 edge of the sterol, which is a likely binding interface with α-helices [36] and minimally perturbs neurosteroid structure [37]. KK123 is, however, an aliphatic diazirine and, as such, may preferentially label nucleophilic amino acids [38]. The two other reagents, KK202 and KK200, incorporate a trifluoromethylphenyl-diazirine (TPD) group at either the 3- or 17-carbon. These were designed to sample the space in the plane of the steroid off either the A-ring (KK202) or the D-ring (KK200). Following UV irradiation, TPD groups generate a carbene which can insert into any bond [39, 40]. Thus, while the TPD groups are bulky and removed several angstroms from the neurosteroid pharmacophore, they should form an adduct precisely at their binding site in the GABA_A_ receptor. Where feasible (KK123, KK202), an alkyne was incorporated in the photolabeling reagents to allow attachment of a fluorophore, purification tag, or an MS reporter tag (*FLI*-tag) via click chemistry [35].

A useful photoaffinity labeling reagent must bind to the same site on a protein as the ligand it mimics and should produce the same effects on protein functions. To determine whether our photoaffinity labeling reagents mimic allopregnanolone as modulators of GABA_A_ receptor function, we assessed modulation of α_1_β_3_ GABA_A_ receptors currents in *Xenopus laevis* oocytes, and enhancement of [^3^H]muscimol binding in human embryonic kidney (HEK) cell membranes expressing α_1_β_3_ GABA_A_ receptors. KK123 enhanced GABA-elicited (0.3 μM) currents 4.2 ± 3.3-fold at 1 μM (*n* = 5 cells) and 8.2 ± 6.7-fold at 10 μM (*n* = 7). KK123 (10 μM) also directly activated α_1_β_3_ GABA_A_ receptors, eliciting 6.3% ± 3.8% (*n* = 5) of the maximum current elicited by a saturating concentration of GABA. KK123 potentiation of GABA-elicited currents and direct activation were absent in α_1_^Q242L^β_3_ GABA_A_ receptors, indicating that KK123 closely mimics the actions of allopregnanolone (the human α_1_^Q242L^ mutation is equivalent to rat α_1_^Q241L^ and is known to selectively prevent neurosteroid action (Fig 1B and Table 1) [14, 27]). KK200 and KK202 also potentiated GABA-elicited currents at 1 and 10 μM and directly activated the channels at 10 μM (Table 1). Positive allosteric modulation by KK202 was somewhat surprising, given that an ether-linked TPD group replaces the 3α-OH group thought to be critical for neurosteroid action [41, 42]. While the effects of KK200 were abolished in α_1_^Q242L^β_3_ receptors, the potentiation by KK202 was reduced by 50% in α_1_^Q242L^β_3_ receptors, suggesting that KK202 may have actions at both the canonical neurosteroid site and other binding sites. Because photolabeling experiments were performed in membranes prepared from cells expressing α_1_β_3_ GABA_A_ receptors, we also examined the ability of the photolabeling reagents to enhance [^3^H]muscimol binding in these membranes. A stable HEK-293 cell line was established with tetracycline-inducible expression of human α_1His-FLAG_ β_3_ GABA_A_ receptors (See Materials and methods); receptor density in these membranes was 20–30 pmol [^3^H]muscimol binding/mg membrane protein. Consistent with previous determinations [43], the average stoichiometry of the receptors was estimated at two α_1_ subunits and three β_3_ subunits using MS label-free quantitation [44] (spectral count). Allopregnanolone enhanced [^3^H]muscimol binding to these recombinant receptors 4-fold with a half maximal effective concentration (EC_50_) of 3.9 ± 5.6 μM (S1 Fig). KK123, KK200, and KK202 all enhanced [^3^H]muscimol binding with EC_50_ values similar to or lower than allopregnanolone (S1 Fig). Collectively, the electrophysiology and radioligand binding data indicate that KK123, KK200, and KK202 are functional mimetics of allopregnanolone.

**Table 1:** AlloP and the photolabeling analogues modulate and activate α_1_β_3_-GABA_A_ receptor currents in *X*. *laevis* oocytes, tested by two-electrode voltage clamp.

**Neurosteroids**	**Potentiation** **1** μ**M NS**	**Potentiation** **10** μ**M NS**	**Direct activation** **10** μ**M NS**
**KK123**	4.2 ± 3.3 (5)	8.2 ± 6.7 (7)	6.3 ± 3.8 (5)
**KK200**	1.6 ± 0.2 (5)	4.9 ± 2.7 (5)	0.14 ± 0.08 (5)
**KK202**	1.4 ± 0.2 (6)	3.9 ± 2.1 (9)	8.97 ± 8.9 (6)
	**Potentiation** **0.1** μ**M AlloP**	**Potentiation** **1** μ**M AlloP**	**Direct activation** **10** μ**M AlloP**
**AlloP**	4.9 ± 1.3 (7)	9.7 ± 4.7 (7)	3.4 ± 1.2 (5)

Potentiation is expressed as potentiation response ratio, calculated as the ratio of the peak response in the presence of GABA and neurosteroids to the peak response in the presence of GABA alone. Direct activation is expressed as percentage of the response to saturating GABA. Data are shown as mean ± SD (number of cells).

**Abbreviation:** AlloP, allopregnanolone; GABA_A_, gamma amino-butyric acid Type A; NS, neurosteroid.

To determine whether KK123—which contains an aliphatic diazirine—photolabels GABA_A_ receptors, we utilized the butynyloxy (alkyne) moiety on KK123 to attach a biotin purification tag for selective enrichment of photolabeled GABA_A_ receptor subunits. HEK-293 cell membranes containing α_1_β_3_ GABA_A_ receptors were photolabeled with 15 μM KK123, solubilized in SDS, and coupled via Cu^2+^-catalyzed cycloaddition to MQ112 (S2A Fig), a trifunctional linker containing an azide group for cycloaddition, biotin for biotin-streptavidin affinity purification, and a cleavable azobenzene group for elution of photolabeled proteins. The photolabeled-MQ112-tagged receptors were bound to streptavidin beads and eluted by cleavage of the linker with sodium dithionite. The purified, photolabeled GABA_A_ receptor subunits were assayed by western blot using anti-α_1_ and anti-β_3_. A band at 52 kDa was observed with both α_1_ and β_3_ subunit antibodies in the KK123 photolabeling group (S2B Fig), indicating that both α_1_ and β_3_ subunits are photolabeled by KK123. In control samples photolabeled with ZCM42—an allopregnanolone photolabeling analogue containing a diazirine at the 6-carbon but no alkyne (S2C Fig)—neither α_1_ nor β_3_ subunits were purified. These data indicated that KK123 can photolabel both α_1_ and β_3_ subunits and is thus an appropriate reagent to use for site identification. A 35 kDa band was intermittently observed in replicate anti-α_1_ western blots (S2B Fig); this is likely to be a proteolytic fragment of the α_1_-subunit that retains the antibody-recognition epitope but was not further analyzed.

### Identification of KK123 photolabeling sites on α_1_ and β_3_ subunits of GABA_A_ receptors

Identification of sterol adducts in hydrophobic peptides has been impeded by multiple challenges, including peptide insolubility during sample digestion, ineffective chromatographic separation of hydrophobic TMD peptides, and neutral loss of sterol adducts from small hydrophobic peptides during ionization and fragmentation. To circumvent these problems, we employed middle-down MS to analyze GABA_A_ receptor TMD peptides and their sterol adducts. This approach identifies each TMD as a single, large peptide and attenuates neutral loss of adduct, facilitating identification of the sites of neurosteroid incorporation. In our studies, α_1His-FLAG_β_3_ GABA_A_ receptors were photolabeled in native HEK cell membranes. The photolabeled proteins were then solubilized in n-dodecyl-β-D-maltoside (DDM)-containing lysis buffer. The pentameric GABA_A_ receptors were purified using anti-FLAG agarose beads, and eluted receptors were digested with trypsin in the presence of the MS-compatible detergent DDM. These conditions generated peptides containing each of the GABA_A_ receptor TMDs in their entirety. The peptides were separated using PLRP-S nano-liquid chromatography and analyzed on a Thermo ELITE orbitrap mass spectrometer. This workflow (S3 Fig) minimized protein/peptide aggregation, simplified MS1-level identification of TMD-sterol adducts, and optimized fragmentation of TMD peptides and their adducts. All eight of the TMD peptides were reliably sequenced with 100% residue-level coverage. In addition, the covalent addition of neurosteroid to the TMD peptides increased the hydrophobicity of TMD peptides and shifted their chromatographic elution to later retention times (S3 Fig). The delayed retention time was used as a critical criterion for identification of photolabeled peptides.

**Fig 2:**
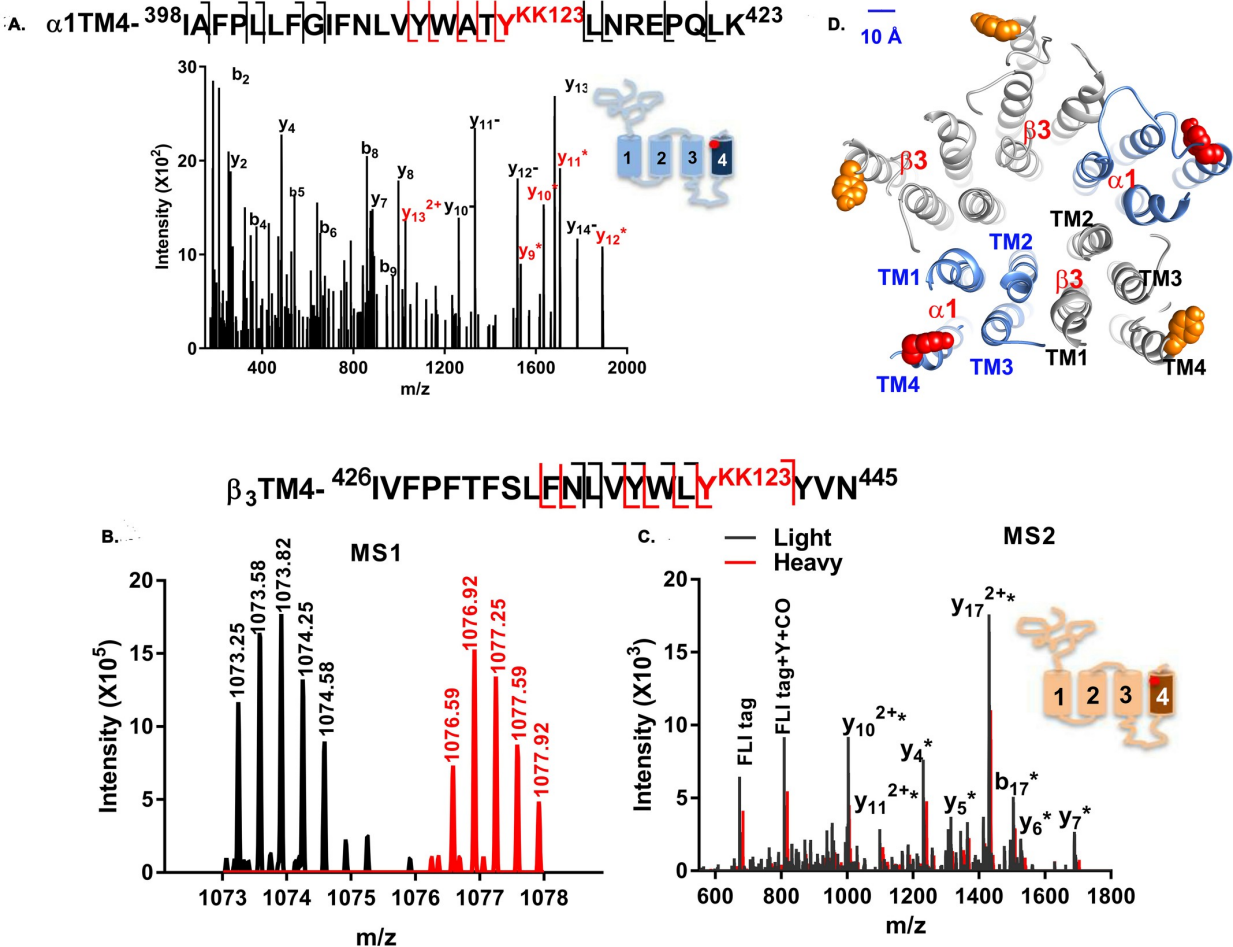
KK123 photolabels α_1_-TM4 and β_3_-TM4 peptides. (A) A representative MS fragmentation spectrum of a KK123 photolabeled α_1_-TM4 peptide (m/z = 875.503, z = 4). The y_9_-y_14_ ions (red) contain the KK123 adduct. The site-defining ions y_8_ and y_9_ indicate that α_1_-Y^415^ (red) was photolabeled by KK123. Fragment ions y_10_^−^ to y_14_^−^ represent neutral loss of the KK123 adduct. (B). MS1 pair of light and heavy form of *FLI*-tag-KK123 photolabeled β_3_ TM4 peptide (m/z = 1,073.246 and m/z = 1,076.580, z = 3). (C) An overlay of light (black) and heavy (red) MS fragmentation spectra of *FLI*-tag-KK123 photolabeled β_3_ TM4 peptide. The KK123 adduct-containing b or y ions are labeled with “*”. Site-defining y_4_ and b_17_ ions identify β_3_-Y^442^ as the photolabeled residue. The photolabeled residues in α_1_- and β_3_-TM4 were identified in three replicate experiments. (D) KK123 photolabeled residues are shown in a homology model of the structure of an α_1_β_3_ GABA_A_ receptor. In the α_1_ subunit, the labeled TM4 tyrosine (red) points toward TM1, whereas in the β_3_ subunit, the labeled tyrosine residue (orange) points toward TM3. The numerical data are included in S2 Data. GABA_A_, gamma amino-butyric acid Type A; MS, mass spectrometry; TM, transmembrane helix.

Two photolabeled peptides were found in the mass spectra of tryptic digests of α_1_β_3_ GABA_A_ receptors photolabeled with KK123 (Fig 2A and S4 Fig). A KK123 adduct of the α_1_-TM4 peptide, ^398^IAFPLLFGIFNLVYWAT**Y**^KK123^LNREPQLK^423^ (m/z = 875.503, z = 4), was identified (add weight of KK123 = 316.27). Site-defining ions in the fragmentation spectra identified the site of KK123 insertion as Y^415^, at the C-terminus of α_1_-TM4 (underlined in the sequence; see Fig 2A). In a separate series of experiments, α_1_β_3_ receptors were photolabeled with KK123, which was then coupled to *FLI*-tag using click chemistry. *FLI*-tag, an azide-containing tag, adds both charge and a heavy/light stable isotope pair to a photolabeled peptide, enhancing identification by creating doublets in the MS1 spectra [35]. MS1 level search for pairs of ions differing by 10.07 mass units found two peptide ion features (m/z = 1,073.246 and m/z = 1,076.580, z = 3) that had identical chromatographic retention times (Fig 2B). Fragmentation spectra revealed both of these peptides as β_3_-TM4 peptide (^426^IVFPFTFSLFNLVYWL**Y**^KK123^YVN^445^) with a KK123-*FLI*-tag adduct (adduct mass = 672.432 and mass = 682.441) on Y^442^ (Fig 2C). In the fragmentation spectrum, ions containing KK123 plus light *FLI*-tag (Fig 2C, black) were different by 10.07 mass units from the corresponding fragment ions from KK123 plus heavy *FLI*-tag (Fig 2C, red), confirming that KK123 photolabels Y^442^ of the β_3_ subunit. β_3_-Y^442^ is located on the C-terminus of β_3_-TM4 in a homologous position to α_1_-Y^415^, the KK123 photolabeling site in α_1_-TM4 (Fig 2D, upper right panel). Thus, KK123 labeling data identified two discrete sites, one in α_1_ and the other in β_3_. We employed additional photolabeling reagents containing TPD groups arrayed around the sterol backbone to confirm whether the KK123-labeled residues represent neurosteroid binding sites and to determine the orientation of the neurosteroids in these sites.

### Photolabeling sites identified by KK200 and KK202

**Fig 3:**
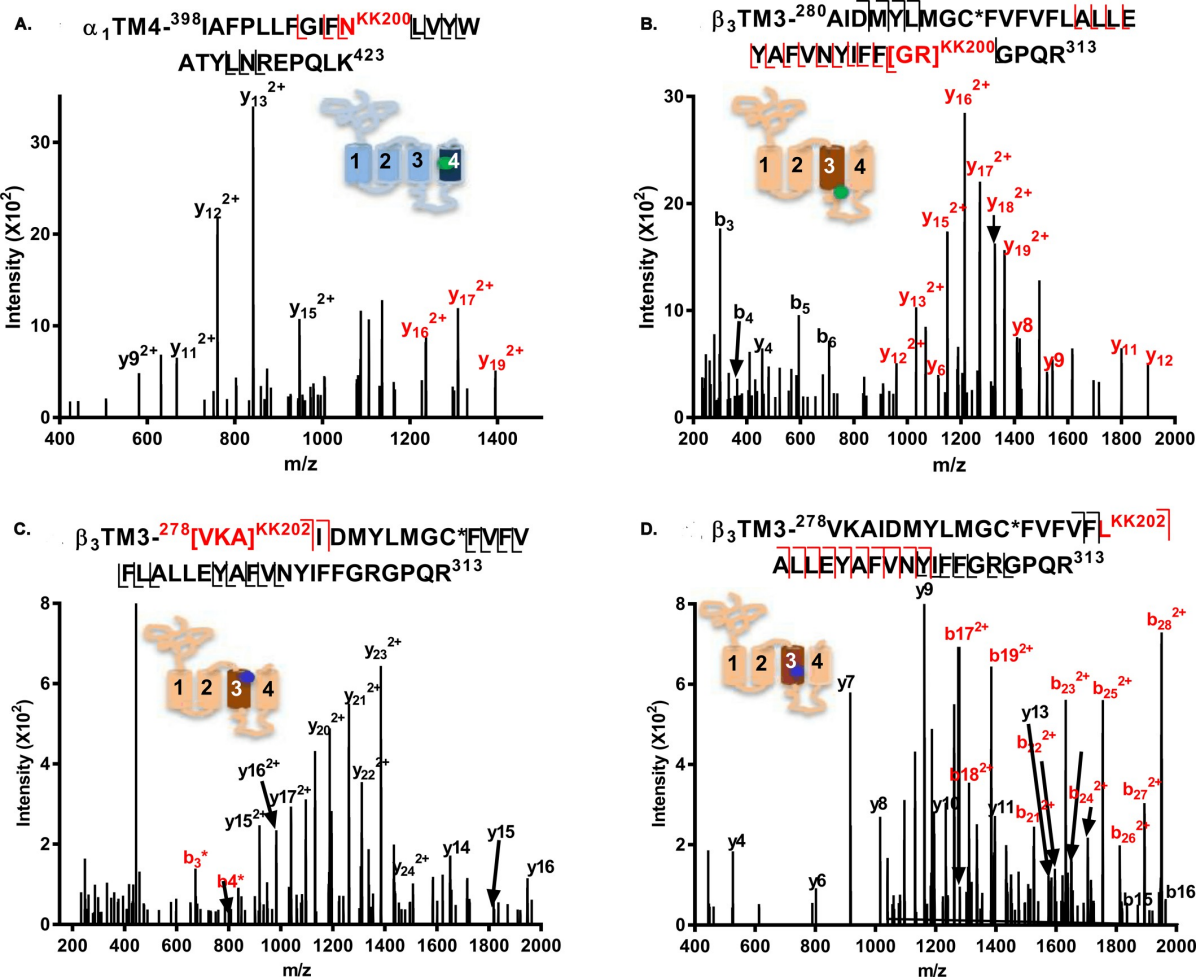
Fragmentation spectra of KK200- and KK202-photolabeled GABA_A_ receptor peptides. (A) An α_1_-TM4 peptide (m/z = 898.002, z = 4) is photolabeled by KK200 at N^408^; (B) A β_3_-TM3 peptide (m/z = 1,188.352, z = 4) is photolabeled by KK200 at [^308^GR^309^]. (C and D) β_3_-TM3 peptides (m/z = 811.453, z = 6) are photolabeled by KK202 at ^226^VKA^228^ (panel C) and L^294^ (panel D). Fragment ions labeled in red contain the neurosteroid adduct. The C* indicates that the cysteine is alkylated by NEM or NEM + DTT. The photolabeled residues shown in panels A–D were all observed in three replicate experiments. The inset schematic of a GABA_A_ receptor subunit in each panel indicates the approximate location of the residues labeled by KK200 (green) and KK202 (blue). The numerical data are included in S3 Data. DTT, 1, 4-dithiothreitol; GABA_A_, gamma amino-butyric acid Type A; NEM, N-ethylmaleimide; TM, transmebrane helix.

KK200, which has a TPD photolabeling group attached at C17 on the steroid backbone, has been previously used to map neurosteroid binding sites on GLIC [31]. Analysis of α_1_β_3_ receptors photolabeled with 15 μM KK200 detected two photolabeled TMD peptides: an α_1_-TM4 peptide, ^398^IAFPLLFGIF**N**^KK200^LVYWATYLNREPQLK^423^, was photolabeled with KK200 (m/z = 898.002; z = 4); site-defining ions in the fragmentation spectra identified N^408^ as the modified residue (Fig 3A). The N^408^ residue (N^407^ in rat) has previously been shown to be critical to neurosteroid potentiation of GABA-elicited currents [14, 15]. A β_3_-TM3 peptide, ^280^AIDMYLMGC^NEM+DTT^FVFVFLALLEYAFVNYIFF**GR**^KK200^GPQR^313^ (*m/z* = 1,188.352; z = 4; N-ethylmaleimide [NEM]; 1, 4-dithiothreitol [DTT]; alkylation adduct), was also photolabeled with KK200. Fragmentation spectra narrowed the possible sites of adduction to G^308^ or R^309^, both at the junction of TM3 with the M3–M4 intracellular loop (Fig 3B).

Analysis of GABA_A_ receptors photolabeled with KK202 (Fig 3C and 3D), identified two photolabeled peptides eluting two minutes apart. Both peptides were identified as the β_3_-TM3 peptide, ^278^**VKA**IDMYLMGC^NEM^FVFVF**L**ALLEYAFVNYIFFGRGPQR^313^ (m/z = 811.453, z = 6). Fragmentation spectra of the earlier eluting peptide localized labeling to a three-residue sequence, ^278^VKA^280^, at the N-terminus of β_3_-TM3 (Fig 3C). The fragmentation spectrum of the later eluting peptide, identified L^294^ as the site of adduction (Fig 3D). (The different retention time of the two photolabeled peptides is likely due to differences in peptide conformation and surface hydrophobicity resulting from incorporation of the photolabeling reagent into different residues.)

### Allopregnanolone prevents photolabeling by neurosteroid analogue photolabeling reagents

**Fig 4:**
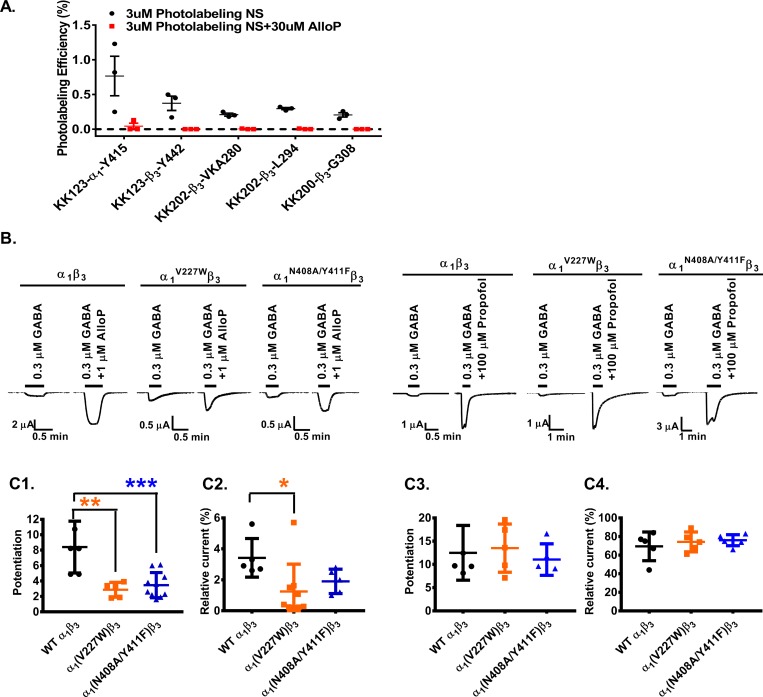
The α_1_ subunit is critical for neurosteroid modulation of α_1_β_3_ GABA_A_ receptors. (A) Photolabeling with 3 μM KK123, KK200, and KK202 (black) is prevented by labeling in the presence of 30 μM allopregnanolone (red). Each circle represents a replicate experiment, and the bars represent mean ± SD, *n* = 3. (B) Sample current traces showing modulation of WT α_1_β_3_, α_1_^V227W^β_3_ and α_1_^N408A/Y411F^β_3_ GABA_A_ receptors by allopregnanolone or propofol. (C1) Allopregnanolone potentiation of GABA-elicited currents is reduced in α_1_^V227W^β_3_ (orange) and α_1_^N408A/Y411F^β_3_ (blue) receptors compared to WT (black). Potentiation is given as the ratio of peak responses to GABA + steroid to GABA alone. Potentiation value of 1 indicates no potentiation by neurosteroids. (C2) Direct activation of α_1_β_3_ GABA_A_ receptors by allopregnanolone is reduced in α_1_^V227W^β_3_ receptors (orange). Direct activation is given in percentage of the peak response to steroid to peak response to saturating GABA + 100 μM propofol. (C3) Potentiation of GABA-elicited currents by propofol, or (C4) direct activation of the receptor in the presence of propofol is not affected by the α_1_^V227W^ or α_1_^N408A/Y411F^ mutations. Each data point represents a replicate experiment. Bars show the mean ± SD (*n* = 6–8). Data were compared by a one-way analysis of variance followed by a Dunnett’s multiple comparison tests of the means. ***p < 0.001; **p < 0.01; *p < 0.05. The numerical data are included in S4 Data. AlloP, allopregnanolone; GABA_A_, gamma amino-butyric acid Type A; WT, wild-type.

An important test of whether the photolabeled sites constitute specific allopregnanolone binding sites is the ability of excess allopregnanolone to competitively prevent photolabeling. Photolabeling studies for site identification were performed using 15 μM photolabeling reagent and achieved levels of labeling efficiency varying from 0.06% to 3.0% (S1 Table). Because allopregnanolone has limited aqueous solubility (about 30 μM) and a large competitor excess is needed to demonstrate competition (particularly with an irreversibly bound ligand), we were limited to studying competition at the photolabeled residues that could be detected following photolabeling at a concentration of 3 μM. Accordingly, we measured the photolabeling efficiency obtained following photolabeling of α_1_β_3_ GABA_A_ receptors with 3 μM KK123, KK200, or KK202 in the presence or absence of 30 μM allopregnanolone. KK123 photolabeled both α_1_-Y^415^ (0.77% efficiency) and β_3_ -Y^442^ (0.37% efficiency). For both of these residues, photolabeling was reduced by >90% in the presence of excess allopregnanolone (Fig 4A). KK200 photolabeled β_3_ -G^308^/R^309^ (0.19% efficiency), and labeling was reduced by 98% in the presence of allopregnanolone. KK202 labeled both β_3_-L^294^ (0.29% efficiency) and β_3_-^278^VKA^280^ (0.21% efficiency) in TM3; labeling of both of these sites was undetectable in the presence of 30 μM allopregnanolone. Studies were also performed to determine whether the orthosteric agonist GABA (1 mM) enhanced photolabeling by 3 uM KK123 or KK200. Labeling efficiency was not significantly enhanced in the presence of GABA. This suggests that there is a small difference in neurosteroid affinity for closed versus open/desensitized states, which is consistent with the fact that neurosteroids have very low efficacy as direct activators of GABA_A_ receptors [45].

### Structural characterization of the photolabeling sites

**Fig 5:**
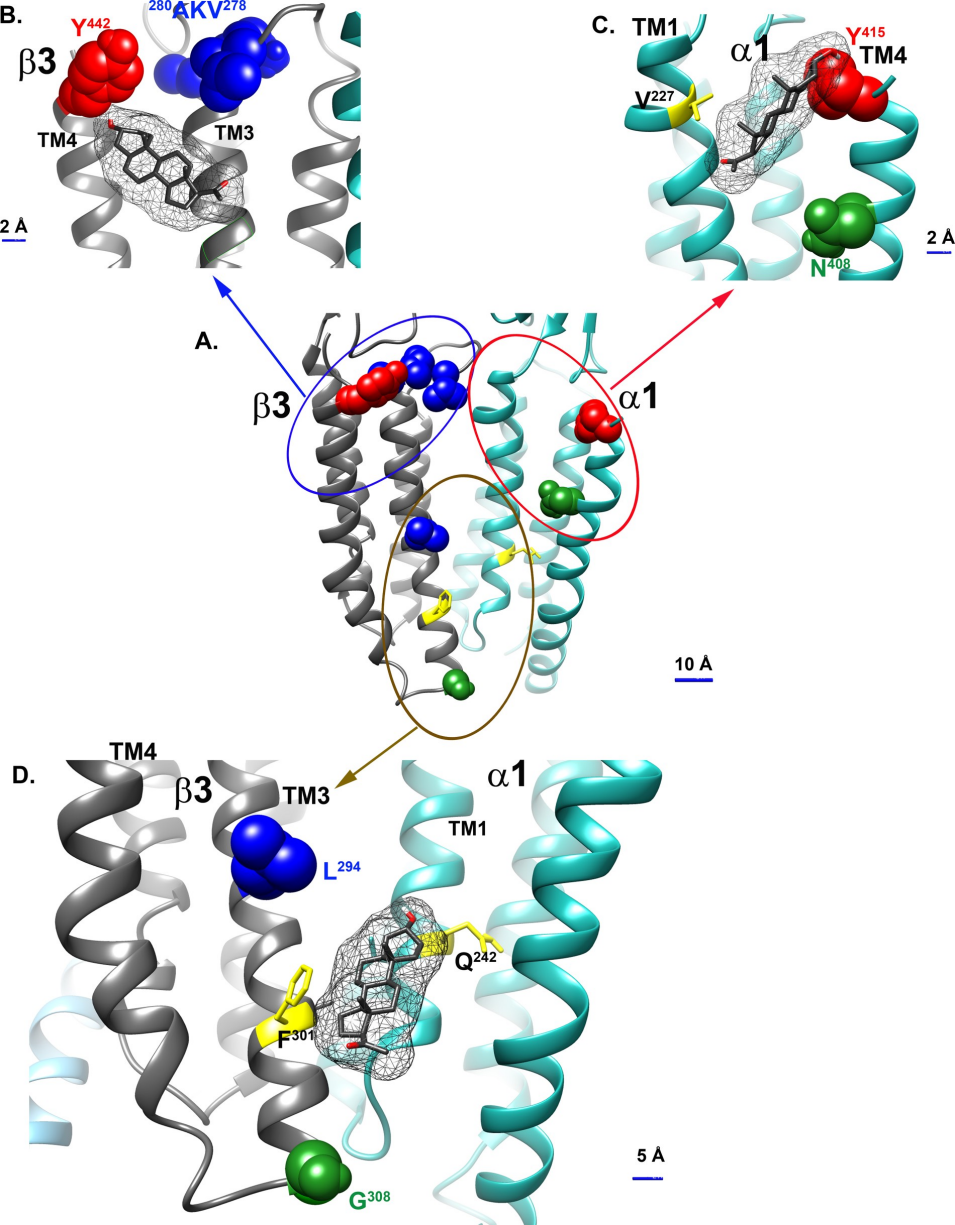
Allopregnanolone docking in the three neurosteroid binding sites identified by photolabeling. (A) The six photolabeling sites identified by photolabeling with KK123, KK200, and KK202 grouped into three clusters: β_3_(+)/α_1_(−) intersubunit sites (brown circle), β_3_ intrasubunit sites (blue circle), and α_1_ intrasubunit sites (red circle); docking of allopregnanolone to the β_3_ intrasubunit site (B), α_1_ intrasubunit site (C), and β_3_(+)/α_1_(−) intersubunit site (D). Residues photolabeled by KK123, α_1_-Y^415^, and β_3_-Y^442^ are colored red; Residues photolabeled by KK200 α_1_-N^408^ and β_3_-G^308^ are colored green; and residues photolabeled by KK202 β_3_-^278^VKA^280^ and L^294^ are colored blue. Residues previously identified as contributing to an intersubunit neurosteroid binding site—α_1_-Q^242^ and β_3_-F^301^—are shown in yellow, as is α_1_-V^227^, a residue in the α_1_ intrasubunit site shown to affect neurosteroid action by site-directed mutagenesis.

Modification of ligand analogues with labeling groups at different locations has been used to determine the orientation of the ligands within their binding pockets [46]. Here, the six residues photolabeled by KK123, KK200, and KK202 were examined in a model of the α_1_β_3_ receptor created by threading the aligned sequence of the α_1_ subunit on the structure of the β_3_ subunit (PDB 4COF) [47]. The photolabeling sites grouped into the following three clusters: cluster 1 (brown circle), β_3_-L^294^ (KK202) and β_3_-G^308^/R^309^ (KK200); cluster 2 (red circle), α_1_-Y^415^ (KK123) and α_1_-N^408^ (KK200); and cluster 3 (blue circle), β_3_-Y^442^ (KK123) and β_3_-^278^VKA^280^ (KK202) (Fig 5A).

In cluster 1 (brown circle, Fig 5A and 5D), β_3_-L^294^ faces into the β(+)/α(−) intersubunit cleft, and G^308^/R^309^ is at the junction between the bottom of TM3 and the TM3–4 intracellular loop. G^308^/R^309^ is two α-helical turns below β_3_-F^301^ (i.e., toward the intracellular terminus of TM3), a residue previously photolabeled by 6-azi pregnanolone in β_3_ homomeric receptors [13]. These data support neurosteroid binding in the β(+)/α(−) interface, consistent with the canonical THDOC and pregnanolone binding sites identified in crystal structures of α_1_(+)/α_1_(−) interfaces in chimeric proteins [16] and in substituted cysteine modification protection studies of α_1_β_2_γ_2_ receptors [18]. The pattern of labeling also indicates that the A-ring of the steroid is oriented upwards in the intersubunit cleft toward the center of the membrane, the D-ring is pointing toward the intracellular termini of the TMDs, and the C5-C6-C7 edge of the steroid is pointing toward the β_3_(+) side of the cleft. Cluster 1 corresponds to a β_3_(+)/α_1_(−) intersubunit site.

In cluster 2 (red circle, Fig 5A and 5C), N^408^ and Y^415^ are both on the C-terminal end of α_1_-TM4, facing toward TM1 within the same α_1_ subunit, consistent with an α_1_ intrasubunit neurosteroid binding site. N^408^, the residue labeled by the C17-TPD of KK200, is two α-helical turns closer to the center of TM4 than is Y^415^, the residue labeled by the C6-diazirine of KK123. This labeling pattern suggests that neurosteroids orient in this site with the A-ring pointing toward the ECD and the D-ring facing to the center of the TMD. Cluster 2 corresponds to an α_1_ intrasubunit site.

In cluster 3 (blue circle, Fig 5A and 5B), Y^442^ is located at the C-terminal end of β_3_-TM4, and ^278^VKA^280^ is located on the TM2–TM3 loop near the extracellular end of β_3_-TM3. The adjacency of these two photolabeling sites suggests an intrasubunit neurosteroid binding site at the extracellular end of β_3_, analogous to the α_1_ intrasubunit site. The labeling of ^278^VKA^280^ in the extracellular loop by the C3-TPD group of KK202 suggests that neurosteroids orient in this site with the A-ring facing the ECD. Cluster 3 corresponds to a β_3_ intrasubunit site.

### Molecular dynamic simulations and docking of neurosteroids

A homology model of the α_1_β_3_ GABA_A_ receptor based on the structure of a β_3_ homomeric GABA_A_ receptor (PDB 4COF) [47] was used to examine the preferred energetic poses of neurosteroid binding to the three binding sites. The homology model was embedded in a 1-palmitoyl-2-oleoyl-sn-glycero-3-phosphocholine (POPC) bilayer and the structure refined by molecular dynamics. We then docked each of the three photoaffinity labeling reagents as well as allopregnanolone to each of the proposed binding sites, using a time course series of snapshots from the simulation trajectory to account for receptor flexibility. All of the neurosteroid photolabeling reagents docked in the three sites; the identified sites are relatively shallow with respect to the protein–lipid interface. Moreover, the neurosteroid analogues were all found to adopt multiple poses in each of the sites with minimal energy differences between the poses (see Materials and methods). Photolabeling data combined with the docking scores (binding energy) and population of a given pose were used to guide selection of the preferred steroid orientation in each site.

**Fig 6:**
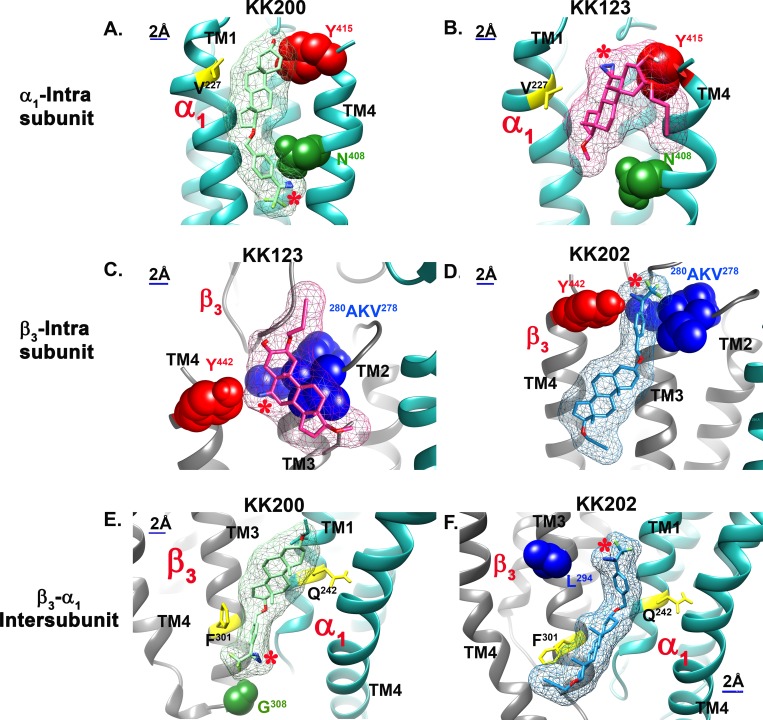
Computational docking of neurosteroid photolabeling analogues to their photolabeling sites on α_1_β_3_-GABA_A_ receptors. (A) KK200 (light green) and (B) KK123 (pink) in α_1_ intrasubunit site. (C) KK123 and (D) KK202 (light blue) in β_3_ intrasubunit site. (E) KK200 and (F) KK202 in β_3_ (+)/-α_1_(−) intersubunit site. The KK123 photolabeled residues α_1_-Y^415^ and β_3_-Y^442^ are colored red, the KK200 photolabeled residues α_1_-N^408^ and β_3_-G^308^ are colored green, and the KK202 β_3_-^278^VKA^280^ and L^294^ are colored blue. The canonical neurosteroid binding site residues Q^242^, F^301^, and the new mutation site V^227^ are colored yellow. The photolabeling diazirine group is indicated with red “*”. GABA_A_, gamma amino-butyric acid Type A.

In the α_1_ intrasubunit site, the poses clustered between TM1 and TM4. The preferred pose (Fig 5C) for allopregnanolone (lowest energy cluster of poses) shows the A-ring oriented toward the ECD with the walls of the predicted binding site lined on one side by N^408^ and Y^415^ and on the other by V^227^. Docking of KK200 in this site has a similar orientation with the A-ring oriented toward the ECD and the TPD group on the D-ring proximal to N^408^ (Fig 6A). Docking of KK-123 shows a preferred pose in which the A-ring is oriented toward the ECD and the C6-diazirine proximal to Y^415^ (Fig 6B). These data elucidate a prior finding that mutations to N^408^ and Y^411^ eliminate potentiation by steroid analogues that lack a hydrogen bonding group on the D-ring [48].

In the β_3_ intrasubunit site, the poses are clustered between TM3 and TM4. Allopregnanolone preferred a pose with the A-ring oriented toward the ECD near Y^442^ and the D-ring proximal to V^290^ (Fig 5B). KK123 was found to dock at the top of the TM helices with the A-ring oriented toward the ECD, placing the 6-diazirine in proximity to Y^442^ (Fig 6C). KK202 was found to dock in a similar orientation but lower in the TM region, with the TPD group in proximity to A^280^ and Y^442^ (Fig 6D).

In the intersubunit site, the preferred pose for allopregnanolone was one of the lowest energy clusters of poses with the A-ring proximal to α_1_-Q^242^ (equivalent to rat α_1_-Q^241^), the D-ring pointing toward the cytoplasmic termini of the TMDs, and the D-ring facing β_3_-F^301^ (Fig 5D). Docking of KK200 showed a similar orientation although shifted slightly upwards toward the ECD, placing the A-ring near α_1_-Q^242^,the benzene ring of the TPD near β_3_-F^301^, and the diazirine in proximity to G^308^ (Fig 6E). The preferred pose of KK202 was closer to TM3 of the β_3_ subunit with the A-ring near α_1_-Q^242^ and the D-ring near the β_3_-F^301^ placing the diazirine in proximity to β_3_-L^294^ (Fig 6F).

The orientation of allopregnanolone docked in our α_1_β_3_ model is nearly identical to the orientation of THDOC in the crystal structure of α1-GLIC [16] (PDB 5OSB). As a confirmation of our docking, we also docked allopregnanolone to the apo-neurosteroid crystal structures of the α_1_-GLIC [16] and α_5_-β_3_ chimeric [17] proteins (PDB 5OSA and PDB 5OJM, respectively) (S5 Fig). The preferred poses for allopregnanolone in the β_3_-α_1_ intersubunit site are nearly identical between the three models. The preferred poses are also very similar in the α_1_ intrasubunit site between our α_1_β_3_ homology model and the structures of the α-homomeric TMDs. The A-ring/D-ring orientation of allopregnanolone in the three neurosteroid sites was consistent with the orientations identified by the photolabeling data in all of the GABA_A_ receptor structures. The calculated binding energies from the docking studies (S4 Table) indicate that the rank order of allopregnanolone affinity for the three sites is β_3_/α_1_ intersubunit site > α_1_ intrasubunit site > β_3_ intrasubunit site.

### Effects of mutations in putative binding sites on neurosteroid action

The β_3_-α_1_ intersubunit binding site identified in our photolabeling studies has been extensively validated by site-directed mutagenesis as a functionally important site. Mutations on the α(−) side of the interface, including Q^241(rat)^L/W and W^246(rat)^L, have been shown to eliminate neurosteroid potentiation and gating of α_1_β_2_γ_2_ GABA_A_ receptors [14, 27, 49]; mutations on the β(+) side of the interface, including F^301^A and L^297^A, have also been shown to partially reduce neurosteroid effect [17]. In the current study, we showed that α_1_^Q242L^β_3_ prevented the action of allopregnanolone, KK123, and KK200 while reducing the effect of KK202 in α_1_β_3_ receptors, confirming the β–α interface as a functionally significant neurosteroid binding site and validating the relevance of our photolabeling reagents (Fig 1B).

Based on computational simulation and docking results, we also identified residues in the proposed α_1_- and β_3_-intrasubunit binding sites that we predicted could be involved in allopregnanolone binding or action (S2 Table and S3 Table for all mutated subunits tested). N^408^ and Y^411^ in α_1_-TM4 line one side of the putative α_1_ intrasubunit site, and V^227^ in α_1_-TM1 lines the other (Fig 5C). α_1_^N407(rat)A^ and α_1_^Y410(rat)W^ mutations have previously been shown to prevent neurosteroid potentiation of GABA-elicited currents in α_1_β_2_γ_2_ GABA_A_ receptors [15]. Our data confirm that the double mutant α_1_^N408A^/^Y411F^β_3_ substantially reduces allopregnanolone potentiation of GABA-elicited currents (Fig 4B and 4C1, ***p < 0.001 versus α_1_β_3_ wild-type). Allopregnanolone (1 μM) potentiation of GABA-elicited currents and direct activation (10 μM) of α_1_^V227W^β_3_ receptors was also significantly reduced in comparison to α_1_β_3_ wild-type (*p < 0.05 and **p < 0.01; Fig 4B, 4C1 and 4C2). To test whether these mutations selectively affected neurosteroid actions, we also compared the effect of propofol in α_1_^V227W^β_3_ and α_1_^N408A/Y411F^β_3_ to its effect on wild-type α_1_β_3_ receptors. Propofol action was not different between the mutant and wild-type receptors, indicating a selective effect on neurosteroid action (Fig 4B, 4C3 and 4C4). The finding that multiple mutations lining the α_1_-intrasubunit binding pocket selectively reduce allopregnanolone action buttresses the evidence that the photolabeled residues identify a specific, functionally important neurosteroid binding site.

Multiple mutations within the putative β_3_-intrasubunit binding site were also tested. However, none of the mutations significantly altered potentiation or activation by allopregnanolone (S2 Table and S3 Table for all of the mutations that were tested). These data suggest that allopregnanolone occupancy of the β_3_ intrasubunit site does not contribute to channel gating. Direct activation of α_1_β_2_^Y284F^γ_2_ receptors by THDOC has previously been shown to be markedly reduced in comparison to wild-type receptors [15], although we found no significant effect of the β_3_-Y^284^ mutation in α_1_β_3_ receptors (S2 Table and S3 Table). The difference in results between experiments in α_1_β_3_ and α_1_β_2_γ_2_ GABA_A_ receptors suggests possible receptor subtype specificity in the functional effects of neurosteroid binding at a β-intrasubunit site.

## Discussion

Collectively, the photolabeling, modeling, and functional data indicate that heteropentameric α_1_β_3_ GABA_A_ receptors contain at least seven binding sites for neurosteroids, of three different types. The use of multiple photolabeling reagents also enabled determination of the orientation of neurosteroids in each proposed class of sites. At least two of these classes are involved in producing the allosteric effect of steroids, the β_3_-α_1_ intersubunit site (two copies per receptor) and the α_1_ intrasubunit site (two copies). Mutations of residues in the proposed β_3_ intrasubunit site (three copies) had no effect on modulation by allopregnanolone although residues were labeled by two photolabeling reagents and labeling was prevented by excess allopregnanolone. Accordingly, the functional significance of this proposed site is not known.

Previous, site-directed mutagenesis studies using electrophysiology readout identified multiple residues, including α_1_-Q^241^, N^407^, Y^410^, T^236^, and β_3_-Y^284^, that selectively contribute to the positive allosteric effects of neurosteroids [14, 27]. Based on homology to the structure of the muscle nicotinic acetylcholine receptor [50], it was hypothesized that there are two neurosteroid binding sites on GABA_A_ receptors: an α_1_-intrasubunit site spanning Q^241^ and N^407^ and an intersubunit site between β_3_-Y^284^ and α_1_-T^236^. Subsequent data [16–18] have clearly established the existence of a β–α intersubunit site. Our photolabeling experiments and homology modeling now show that the previously identified residues contribute to multiple distinct neurosteroid binding sites, albeit differently than originally proposed. It is noteworthy that the α_1_-intrasubunit site was not identified in the X-ray crystallographic structures of α_1_-GLIC chimeras bound with THDOC or the α_5_-β_3_ chimera bound with pregnanolone. This is likely because the proteins with steroid bound in the intrasubunit site did not form stable crystals.

Mutations in either the β_3_-α_1_ intersubunit site or the α_1_-intrasubunit site can ablate both potentiation and direct activation by allopregnanolone, indicating that these are not distinct sites mediating potentiation and direct activation. The data also do not conform to simple energetic additivity for the two sites. The observation that mutations in either binding site can largely eliminate neurosteroid effect indicates that these two sites do not function completely independently and suggests allosteric interaction between the two sites. Development of site-selective neurosteroid analogues (PAMs and antagonists) should facilitate clarification of the mechanisms of allosteric interaction between these two sites.

In light of the demonstration of multiple neurosteroid binding sites in α_1_β_3_ GABA_A_ receptors, the possibility of additional isoform-specific sites must be considered. The strong sequence homology between the TMDs of the six α-subunits and three β-subunits suggests that there will not be large isoform differences in the intersubunit site [27]. In contrast, the contribution of ECD residues to the α- and β-intrasubunit sites suggests possible isoform-specific differences. The sequence homology between the γ and δ subunits and α and β subunits suggests that there may also be intrasubunit neurosteroid binding sites in these isoforms. Identification of a neurosteroid binding site on a δ-subunit would be of particular relevance because GABA_A_ receptors containing δ-subunits are particularly sensitive to neurosteroids [51–53]. High-resolution, cryo-electron microscopy structures of α_1_β_3_γ_2_ GABA_A_ receptors [54–56] have been published since initial submission of this work. The structural homology between γ_2_ subunits and α and β subunits suggests that there may also be intrasubunit neurosteroid binding sites in the γ_2_ subunit. The existence of multiple sites in which neurosteroids bind with different orientation may also offer some explanation for the difficulty in identifying neurosteroid antagonists [57] and for the differences in single-channel electrophysiological effects of various neurosteroid analogues [28, 30]. The possibility of multiple isoform-specific sites with distinct patterns of neurosteroid affinity, binding orientation, and effect offers the exciting potential for the development of isoform-specific agonists, partial agonists, and antagonists with targeted therapeutic effects.

## Materials and methods

### cDNA constructs

The human α_1_ and β_3_ subunits were subcloned into pcDNA3 for molecular manipulations and cRNA synthesis. Using QuikChange mutagenesis (Agilent), a FLAG tag was first added to the α_1_ subunit then an 8xHis tag was added to generate the following His-FLAG tag tandem (QPSLHHHHHHHHDYKDDDDKDEL), inserted between the fourth and fifth residues of the mature peptide. The α1 and β3 subunits were then transferred into the pcDNA4/TO and pcDNA5/TO vectors (ThermoFisher Scientific, Waltham, MA), respectively, for tetracycline-inducible expression. For *X*. *laevis* oocytes, point mutations were generated using the QuikChange site-directed mutagenesis kit (Agilent Technologies, Santa Clara, CA) and the coding region fully sequenced prior to use. The cDNAs were linearized with Xba I (NEB Labs, Ipswich, MA), and the cRNAs were generated using T7 mMessage mMachine (Ambion, Austin, TX).

### Cell culture

The tetracycline-inducible cell line HEK T-Rex^TM^-293 (ThermoFisher) was cultured under the following conditions: cells were maintained in DMEM/F-12 50/50 medium containing 10% fetal bovine serum (tetracycline-free, Takara, Mountain View, CA), penicillin (100 units/ml), streptomycin (100 g/ml), and blastcidine (2 μg/ml) in a humidified atmosphere containing 5% CO_2_. Cells were passaged twice each week, maintaining subconfluent cultures. Stably transfected cells were cultured as above with the addition of hygromycin (50 μg/ml) and Zeocin (20 μg/ml).

### Generation of high-expression stable cell line

A stable cell line was generated by transfecting HEK T-Rex^TM^-293 cells with human α_1_-8x His-FLAG pcDNA4/TO and human β_3_ pcDNA5/TO in a 150 mm culture dish, using the Effectene transfection reagent (Qiagen). Two days after transfection, selection of stably transfected cells was performed with hygromycin and zeocin until distinct colonies appeared (usually after two weeks). Medium was exchanged several times each week to maintain antibiotic selection.

Individual clones (about 65) were selected from the dish and transferred to 24-well plates for expansion of each clone selected. When the cells grew to a sufficient number, about 50% confluency, they were split into two other plates, one for a surface ELISA against the FLAG epitope and a second for protein assay, to normalize surface expression to cell number [58]. The best eight clones were selected for expansion into 150 mm dishes, followed by [^3^H]muscimol binding. Once the best expressing clone was determined, the highest-expressing cells of that clone were selected through fluorescence-activated cell sorting (FACS).

### FACS

FACS was done against the FLAG epitope, using a phycoerythrin (PE)-conjugated anti-FLAG antibody. Fluorescent-activated cells (1 ml containing about 10 million cells) were sorted on the AriaII cell sorter (Washington University Pathology Core), collecting 0.5% of the highest-fluorescing cells in a culture tube containing complete medium. The cells were plated in a 35 mm dish and expanded until a near confluent 150 mm dish was obtained. Cells were enriched for expression by FACS three times. A final FACS was performed to select individual cells into a 96-well plate, which resulted in only 10 colonies of cells. These colonies were expanded and assayed for [^3^H]muscimol binding; the highest-expressing clone was used for experiments.

### Induction of GABA_A_ receptor expression

Stably transfected cells were plated into fifty 150 mm dishes. After reaching 50% confluency, GABA receptors were expressed by inducing cells with 1 μg/ml of doxycycline with the addition of 5 mM sodium butyrate. Cells were harvested after 48 to 72 hours after induction.

### Membrane protein preparation

HEK cells, after tetracycline induction, grown to 70%–80% confluency, were washed with 10 mM sodium phosphate/proteinase inhibitors (Sigma-Aldrich, St. Louis, MO) two times and harvested with cell scrapers. The cells were washed with 10 mM sodium phosphate/proteinase inhibitors and collected by centrifugation at 1,000 g at 4°C for 5 minutes. The cells were homogenized with a glass mortar Teflon pestle for 10 strokes on ice. The pellet containing the membrane proteins was collected after centrifugation at 34,000 g at 4°C for 30 minutes and resuspended in a buffer containing 10 mM potassium phosphate and 100 mM KCl. The protein concentration was determined with micro-BCA protein assay and stored at −80°C.

### [^3^H]muscimol binding

[^3^H]muscimol binding assays were performed using a previously described method with minor modification [59]. Briefly, HEK cell membranes proteins (50 μg/ml final concentration) were incubated with 1–2 nM [^3^H]muscimol (30 Ci/mmol; PerkinElmer Life Sciences), neurosteroid in different concentrations (1 nM-10 μM), binding buffer (10 mM potassium phosphate, 100 mM KCl [pH 7.5]), in a total volume of 1 ml. Assay tubes were incubated for 1 hour at 4°C in the dark. Nonspecific binding was determined by binding in the presence of 1 mM GABA. Membranes were collected on Whatman/GF-C glass filter paper using a Brandel cell harvester (Gaithersburg, MD). To determine the Bmax of [^3^H]muscimol binding, 100 μg/ml of proteins were incubated with 250 nM [^3^H]muscimol, with specific activity reduced to 2 Ci/mmol, for 1 hour at 4°C in the dark. The membranes were collected on Whatman/GF-B glass filter papers using manifold. Radioactivity bound to the filters was measured by liquid scintillation spectrometry using Bio-Safe II (Research Products International Corporation). Each data point was determined in triplicate.

### Photolabeling of α_1_β_3_ GABA_A_ receptor

For all the photolabeling experiments, 10–20 mg of HEK cell membrane proteins (about 300 pmol [^3^H]muscimol binding) were thawed and resuspended in buffer containing 10 mM potassium phosphate, 100 mM KCl (pH 7.5) at a final concentration of 1.25 mg/ml. For photolabeling site identification experiments, 15 μM neurosteroid photolabeling reagent was added to the membrane proteins and incubated on ice for 1 hour. For the photolabeling competition experiments, 3 μM neurosteroid photolabeling reagent in the presence of 30 μM allopregnanolone or the same volume of ethanol was added for incubation. The samples were then irradiated in a quartz cuvette for 5 minutes, by using a photoreactor emitting light at >320 nm [59]. The membrane proteins were then collected by centrifugation at 20,000 g for 45 minutes. All of the photolabeling experiments to identify sites of neurosteroid photolabeling were performed at least three times. The photolabeled peptides and residues described in the text were all observed in replicate experiments.

### Cycloaddition (click reaction) of *FLI*-tag to KK123-photolabeled proteins

The amount of 10 mg of KK123 or ZCM42 photolabeled HEK membrane proteins were solubilized in 1 ml 2% SDS/PBS and incubated at room temperature for 2 hours. The protein lysate was collected by centrifugation at 21,000 g for 30 minutes. *FLI*-tag was clicked to the KK123- or ZCM-photolabeled proteins at room temperature overnight in PBS buffer containing 2% SDS, 100 μM FLI-tag [35], 2.5 mM sodium ascorbate, 250 μM Tris [(1-benzyl-1H-1,2,3triazol-4-yl)methyl]amine, and 2.5 mM CuSO_4_. The amount of 1% Triton/PBS was added to the protein lysate to an SDS final concentration of 0.05%. The protein lysate was loaded onto a streptavidin agarose column. The flow through was reloaded to the column two times or till the flow through was colorless and the streptavidin column was dark orange yellow. The column was washed with 10 ml 0.05% Triton/PBS and eluted by 10 ml 100 mM sodium dithionite/0.05%Triton/PBS. The column was turned into colorless after elutions. The eluted proteins were concentrated into 100 μl with 30 kDa cutoff Centricon apparatus. The supernatant of the Centricon tube was added into SDS-sample loading buffer, loaded to a 10% SDS-PAGE, and transferred to a PVDF membrane, followed by western blot with polyclonal rabbit anti-α_1_ raised against a peptide mapping within a cytoplasmic domain of human GABAR α_1_ subunit [60] (Santa Cruz Biotechnology) or monoclonal anti-β_3_ antibody against 370–433 of mouse GABAR β_3_ subunit [61] (NeuroMab).

### Purification of α_1_β_3_ GABA_A_ receptors

The photolabeled membrane proteins were resuspended in lysis buffer containing 1% DDM, 0.25% cholesteryl hemisuccinate (CHS), 50 mM Tris (pH 7.5), 150 mM NaCl, 2 mM CaCl_2_, 5 mM KCl, 5 mM MgCl_2_, 1 mM EDTA, and 10% glycerol at a final concentration of 1 mg/ml. The membrane protein suspension was homogenized using a Teflon pestle in a motor-driven homogenizer and incubated at 4°C overnight. The protein lysate was centrifuged at 20,000 g for 45 minutes, and supernatant was incubated with 0.5 ml anti-FLAG agarose (Sigma) at 4°C for 2 hours. The anti-FLAG agarose was then transferred to an empty column, followed by washing with 20 ml washing buffer (50 mM triethylammonium bicarbonate and 0.05% DDM). The GABA_A_ receptors were eluted with ten 1-ml 200 μg/ml FLAG peptide and 100 μg/ml 3X FLAG (ApexBio) in the washing buffer. The 10 ml effective elutions containing GABA_A_ receptors (tested by western blot with anti-α1 or anti-β_3_ antibody) were concentrated by 100 kDa cutoff Centricon filters into 0.1 ml.

### Middle-down MS

The purified GABA_A_ receptors (100 ul) were reduced by 5 mM tris (2-carboxyethyl) phosphine (TCEP) at for 30 minutes followed by alkylation with 7.5 mM NEM for 1 hour in the dark. The NEM was quenched by 7.5 mM DTT for 15 minutes. These three steps were done at room temperature. Eight μg of trypsin was added to the protein samples and incubated at 4°C for 7–10 days. The digest was terminated by adding formic acid (FA) in a final concentration of 1%. The samples were then analyzed by an OrbiTrap ELITE mass spectrometer (ThermoFisher) as in previous work [13, 31] with some modifications. Briefly, a 20 μl aliquot was injected by an autosampler (Eksigent) at a flow rate of 800 nl/min onto a home-packed polymeric reverse phase PLRP-S column (Agilent, 12 cm × 75 μm, 300 Å). An acetonitrile (ACN) 10%–90% concentration gradient was applied in the flow rate of 800 nl/min for 145 minutes to separate peptides. Solvent A was 0.1% FA/water, and solvent B was 0.1%FA/ACN. The ACN gradient was as follows: isocratic elution at 10% solvent B, 1–60 minutes; 10%–90% solvent B, 60–125 minutes; 90% solvent B, 125–135 minutes; 90%–10% solvent B, 135–140 minutes; isocratic solvent B, 140–145 minutes. For the first 60 minutes, a built-in divert valve on the mass spectrometer was used to remove the hydrophilic contaminants from the mass spectrometer. The survey MS1 scans were acquired at acquired at high resolution (60,000 resolution) in the range of m/z = 100–2,000, and the fragmentation spectra were acquired at 15,000 resolution. Data-dependent acquisition of the top 20 MS1 precursors with exclusion of singly charged precursors was set for MS2 scans. Fragmentation was performed using collision-induced dissociation or high-energy dissociation with normalized energy of 35%. The data were acquired and reviewed with Xcalibur 2.2 (ThermoFisher). The MS experiments of identification of the photolabeling sites and competition of photolabeling were replicated at least three times.

### MS data processing and analysis

The LC-MS data were searched against a customized database containing the sequence of the GABA_A_ receptor 8X His-FLAG-α_1_ and β_3_ subunit and filtered with 1% false discovery rate using PEAKS 8.5 (Bioinformatics Solutions Inc.). Search parameters were set for a precursor mass accuracy of 30 ppm, fragmentation ion accuracy of 0.1 Da, up to three missed cleavage on either side of peptide with trypsin digestion. Methionine oxidation, cysteine alkylation with NEM and DTT, any amino acids with adduct of KK123 (mass = 372.16), KK200 (mass = 462.27), KK202 (mass = 500.31), KK123 with light *FLI*-tag (mass = 672.4322), and KK123 with heavy *FLI*-tag (mass = 682.44) were included as variable modification.

### Receptor expression in *X*. *laevis* oocytes

The GABA_A_ receptors were expressed in oocytes from the African clawed frog (*X*. *laevis*). Frogs were purchased from Xenopus 1 (Dexter, MI) and housed and cared for in a Washington University Animal Care Facility under the supervision of the Washington University Division of Comparative Medicine. Harvesting of oocytes was conducted under the Guide for the Care and Use of Laboratory Animals as adopted and promulgated by the National Institutes of Health. The animal protocol was approved by the Animal Studies Committee of Washington University in St. Louis (approval No. 20170071).

The oocytes were injected with a total of 12 ng cRNA in 5:1 ratio (α_1_:β_3_) to minimize the expression of β_3_ homomeric receptors. Following injection, the oocytes were incubated in ND96 with supplements (96 mM NaCl, 2 mM KCl, 1.8 mM CaCl_2_, 1 mM MgCl_2_, 2.5 mM Na pyruvate, 5 mM HEPES, and 100 U/ml + 100 μg/ml penicillin + streptomycin and 50 μg/ml gentamycin [pH 7.4]) at 16°C for 1–2 days prior to conducting electrophysiological recordings.

### Electrophysiological recording

The electrophysiological recordings were conducted using standard two-electrode voltage clamp. Borosilicate capillary glass tubing (G120F-4, OD = 1.20 mm, ID = 0.69 mm; Warner Instruments, Hamden, CT) were used for voltage and current electrodes. The oocytes were clamped at −60 mV. The chamber (RC-1Z; Warner Instruments, Hamden, CT) was perfused with ND96 at 5–8 ml min^−1^. Solutions were gravity-applied from 30-ml glass syringes with glass luer slips via Teflon tubing.

The current responses were amplified with an OC-725C amplifier (Warner Instruments), digitized with a Digidata 1200 series digitizer (Molecular Devices) and were stored using pClamp (Molecular Devices). The peak amplitude was determined using Clampfit (Molecular Devices).

The stock solution of GABA was made in ND96 bath solution at 500 mM, stored in aliquots at −20°C, and diluted as needed on the day of experiment. Stock solution of propofol (200 mM in DMSO) was stored at room temperature. The steroids were dissolved in DMSO at 10 mM and stored at room temperature.

### Electrophysiology data analysis

The α_1_β_3_ wild-type and mutant receptors were tested (see Table 1 and S2 and S3 Tables) for potentiation by steroids (3α5α-allopregnanolone, 3α5β-pregnanolone, KK123, KK200, and KK-202) and direct activation by steroids (allopregnanolone KK123, KK200, KK-202, and pregnanolone). As control, several receptor isoforms were tested for potentiation by propofol. For each receptor type, we also determined constitutive open probability (P_o,const_).

To estimate P_o,const_, the effect of 100 μM picrotoxin (estimated P_o_ = 0) on the holding current was compared to the peak response to saturating GABA + 100 μM propofol (estimated P_o_ = 1). P_o,const_ was then calculated as I_picrotoxin_ ÷ (I_picrotoxin_ − I_GABA+propofol_) [62].

Potentiation is expressed as the potentiation response ratio, calculated as the ratio of the peak response to GABA + modulator (steroid or propofol) to the peak response to GABA alone. The concentration of GABA was selected to produce a response of 5%–15% of the response to saturating GABA + 100 μM propofol.

Direct activation by steroids was evaluated by comparing the peak response to 10 μM neurosteroid to the peak response to saturating GABA + 100 μM propofol. Direct activation by steroids is expressed in units of open probability that includes constitutive open probability. All data are given as mean ± SD and analyzed by one-way ANOVA followed by Dunnet’s multiple comparison to the control wild-type group.

### Docking simulations

A homology model of the α_1_β_3_ GABA_A_ receptor was developed using the crystal structure of the human β_3_ homopentamer published in 2014 (PDB ID: 4COF) [47]. In this structure, the large cytoplasmic loops were replaced with the sequence SQPARAA used by Jansen and colleagues [63] The pentamer subunits were organized as A α_1_, B β_3_, C α_1_, D β_3_, E β_3_. The α_1_ sequence was aligned to the β_3_ sequence using the program MUSCLE [64]. The pentameric alignment was then used as input for the program Modeller [65], using 4COF as the template; a total of 25 models were generated. The best model as evaluated by the DOPE score [66] was then oriented into a POPC membrane, and the system was fully solvated with 40715 TIP3 water molecules and ionic strength set to 0.15 M KCl. A 100 ns molecular dynamics trajectory was then obtained using the CHARMM36 force field and NAMD. The resulting trajectory was then processed using the utility mdtraj [67], to extract a snapshot of the receptor at each nanosecond of time frame. These structures were then mutually aligned by fitting the alpha carbons, providing a set of 100 mutually aligned structures used for docking studies.

The docking was performed using AutoDock Vina [68] on each of the 100 snapshots in order to capture the receptor flexibility. Docking boxes were built for the β_3_ intrasubunit site (cluster 3), the α_1_ intrasubunit site (cluster 2), and the β_3_-α_1_ intersubunit site (cluster 1). The boxes were centered around the residues photolabeled by KK123, KK200, and KK202 and had dimensions of 25 × 25 × 25 Ångströms, large enough to easily fit the linear dimensions of all of the steroids. For docking studies of allopregnanolone, the docking boxes were placed in the same locations but had smaller dimensions of 20 × 20 × 20 Ångströms. Docking was limited to an energy range of 3 kcal from the best docking pose and was limited to a total of 20 unique poses. The docking results for a given site could result in a maximum of 2,000 unique poses (20 poses × 100 receptor structures); these were then clustered geometrically using the program DIVCF [69]. The resulting clusters were then ranked by Vina score and cluster size and visually analyzed for compatibility with the photolabeling results, which is the photolabeling group oriented in the correct direction to produce the observed photo adducts.

### Chemicals

The inorganic salts used in the buffers, GABA, picrotoxin, and the steroids 3α, 5α-allopregnanolone, and 3α,5β-pregnanolone were purchased from Sigma-Aldrich. Propofol was purchased from MP Biomedicals (Solon).

## Supporting information

S1 Fig Enhancement of [^3^H]-muscimol binding to α_1_β_3_ GABA_A_ receptors by allopregnanolone and its photolabeling analogues. The EC_50_ values (in μM) are 3.9 ± 5.7 (*n* = 9) for allopregnanolone; 1.6 ± 0.2 (*n* = 9) for KK123; 0.54 ± 0.18 (*n* = 9) for KK200; and 1.1 ± 0.27 (*n* = 9) for KK202. The numerical data are included in S5 Data. EC_50_, half maximal effective concentration.
(PPTX)

S2 Fig Purification of KK123 photolabeled α_1_β_3_ GABA_A_ receptors via a trifunctional linker MQ112. (a) The structure of MQ112. (b) Purification of KK123 photolabeled GABA_A_ receptor α_1_ and β_3_ subunit by MQ112, via a click reaction, visualized by western blot with anti-α_1_ and anti-β_3_. (c) The structure of ZCM42.
(PPTX)

S3 Fig Workflow for identifying neurosteroid photolabeling sites in GABA_A_ receptors. (PPTX)

S4 Fig Fragmentation spectrum of KK123 photolabeled β_3_TM4 peptide. Y_4_ and b_17_ (in red) fragment ions containing a KK123 adduct indicate that Y^442^ is photolabeled by KK123. The fragment ions with neutral loss of the adduct are labeled as b_17_*^2+^, b_18_*^2+^, b_19_*^2+^, and y_20_*^2+^. The numerical data are included in S6 Data.
(PPTX)

S5 Fig Computational docking of allopregnanolone to the putative intersubunit and intrasubunit neurosteroid sites on α_1_β_3_-GABA_A_ receptors, α_5_-β_3_ chimeric receptors (PDB 5OJM), and α_1_-GLIC chimeric receptors (PDB 5OSA). Each panel shows the structural model of an α_1_β_3_ GABA_A_ receptor (based on the structure of the β_3_ homomeric GABA_A_ receptor [PDB 4COF] superimposed on models based on the X-ray crystallographic structures of the α_5_-β_3_ chimera (panels a and c) or the α_1_-GLIC chimera (panels b and d). Docking of allopregnanolone to β_3_-α_1_ intersubunit site is shown above (panels a and b) and to the α_1_ intrasubunit site below (panels c and d). The β_3_ subunit in the α_1_β_3_ GABA_A_ receptor model is colored light gray, and the α_1_ subunit is colored dark gray. TMDs in the α_5_-β_3_ chimera (panels a and c) are colored gold; TMDs in the α_1_-Glic chimera (panels b and d) are pink. The preferred pose of allopregnanolone docking to the α_1_β_3_-GABA_A_ receptors is shown in cyan and to the chimeric receptors in dark red. The KK123 photolabeled residue α_1_-Y^415^ is colored in red, and the KK200 photolabeled residue α_1_-N^408^ is colored green. The canonical neurosteroid binding residues Q^242^, F^301^, and the new mutation residue—V^227^—are colored yellow. GLIC, *Gloeobacter* ligand-gated ion channel; TMD, transmembrane domain.
(PPTX)

S1 Table Photolabeling efficiency of the photolabeling analogues (15 μM) in each of the identified sites. Data are presented as area under the curve of selected ion chromatograms of photolabeled peptides as a percentage of the area under the curve of corresponding nonphotolabeled peptides.
(DOCX)

S2 Table Potentiation of α_1_β_3_ receptors GABA_A_ receptors in *X*. *laevis* oocytes. Potentiation is expressed as potentiation response ratio, calculated as the ratio of the peak responses in the presence of GABA and neurosteroids to the peak response in the presence of GABA alone. The GABA concentrations were selected to generate a response of 5%–15% of the response to saturating GABA. Data are shown as mean ± SD (number of cells). One-way ANOVA followed by Dunnett’s multiple comparison to the control wild-type group was used for statistical analysis. *p < 0.05; **p < 0.01; and ***p < 0.001.
(DOCX)

S3 Table Direct activation of α_1_β_3_ GABA_A_ receptors in *X*. *laevis* oocytes by 10 μM allopregnanolone (3α5αP) and pregnanolone (3α5βP). Direct activation is expressed in units of open probability. Data are shown as mean ± SD (number of cells). One-way ANOVA followed by Dunnett’s multiple comparison to the control wild-type group was used for statistical analysis. *p < 0.05 and ***p < 0.001.
(DOCX)

S4 Table Docking results for S5 Fig. Free energies of binding are predicted by Vina and are in kilocalories per mole.
(DOCX)

S1 Data The numerical data for construction of Fig 1B. Table of GABA-elicited currents (in μA) in numerical form in wild-type α_1_β_3_ GABA_A_ receptors and mutant α_1_^Q242L^β_3_ receptors in the presence or absence of allopregnanolone, KK123, KK200, and KK202.
(XLSX)

S2 Data The numerical data for the MS spectra in Fig 2. Each set of data represents an MS spectrum of a KK123-photolabeled peptide in numerical form with m/z of ions and their corresponding ion intensity listed in columns. MS, mass spectrometry.
(XLSX)

S3 Data The numerical data for the MS fragmentation spectra in Fig 3. Each set of data represents an MS fragmentation spectrum of a KK200- or KK202-photolabeled peptide in numerical form. The m/z of fragmentation ions and their corresponding ion intensity are listed in columns. MS, mass spectrometry.
(XLSX)

S4 Data The numerical data for construction of Fig 4. Fig 4A. Summary of the photolabeling efficiency of different peptides by KK123, KK200, or KK20 in the presence or absence of 30 μM allopregnanolone, from three replicate experiments. Fig 4B. The magnitude of GABA-elicited currents (in μA) recorded from wild-type α_1_β_3_ GABA_A_ receptors, mutant α_1_^V227W^β_3_, or α_1_^N408A/Y411F^β_3_ receptors in the presence or absence of allopregnanolone or propofol. Fig 4C. Summary of potentiation or direct activation of wild-type α_1_β_3_ GABA_A_ receptors, mutant α_1_^V227W^β_3_, or α_1_^N408A/Y411F^β_3_ receptors by allopregnanolone or propofol in replicate samples.
(XLSX)

S5 Data Summary of data showing enhancement of [^3^H]-muscimol binding to α_1_β_3_ GABA_A_ receptors by different neurosteroids in S1 Fig. Each data point represents an individual replicate expressed as percentage of control. Control is defined as the average of the replicate values obtained with 0 μM neurosteroid.
(XLSX)

S6 Data The numerical data for the MS fragmentation spectrum of the KK123-photolabeled β_3_-TM4 peptide in S4 Fig. The table lists the fragmentation ions (m/z) and their corresponding ion intensities in the spectrum. MS, mass spectrometry; TM, transmembrane.
(XLSX)

S1 Text The material and methods for chemical synthesis and verification of structure of novel neurosteroid photolabeling analogues (KK200 and KK202) and the trifunctional linker (MQ112). (DOCX)

## Abbreviations

ACN: acetonitrile
CHS: cholesteryl hemisuccinate
CNS: central nervous system
DDM: n-dodecyl-β-D-maltoside
DTT: 1, 4-dithiothreitol
EC_50_: half maximal effective concentration
ECD: extracellular domain
FA: formic acid
FACS: fluorescence-activated cell sorting
GABA: gamma amino-butyric acid
GABA_A_ receptor: gamma amino-butyric acid Type A receptor
GLIC: Gloeobacter ligand-gated ion channel
HEK: human embryonic kidney
MS: mass spectrometry
NEM: N-ethylmaleimide
PAM: positive allosteric modulator
pLGIC: pentameric ligand-gated ion channel
POPC: 1-palmitoyl-2-oleoyl-sn-glycero-3-phosphocholine
TBPS: [^35^S]t-butylbicyclophosphorothionate
TCEP: tris (2-carboxyethyl) phosphine
THDOC: tetrahydroxy-desoxycorticosterone
TMD: transmembrane domain
TPD: trifluoromethylphenyl-diazirine
