# Supplementary figures and images for: Multiple functional neurosteroid binding sites on GABAA receptors
Source: PLoS Biol. 2019 Mar 7;17(3):e3000157. doi: 10.1371/journal.pbio.3000157 (PMC6424464; doi:10.1371/journal.pbio.3000157)

## Slide 1
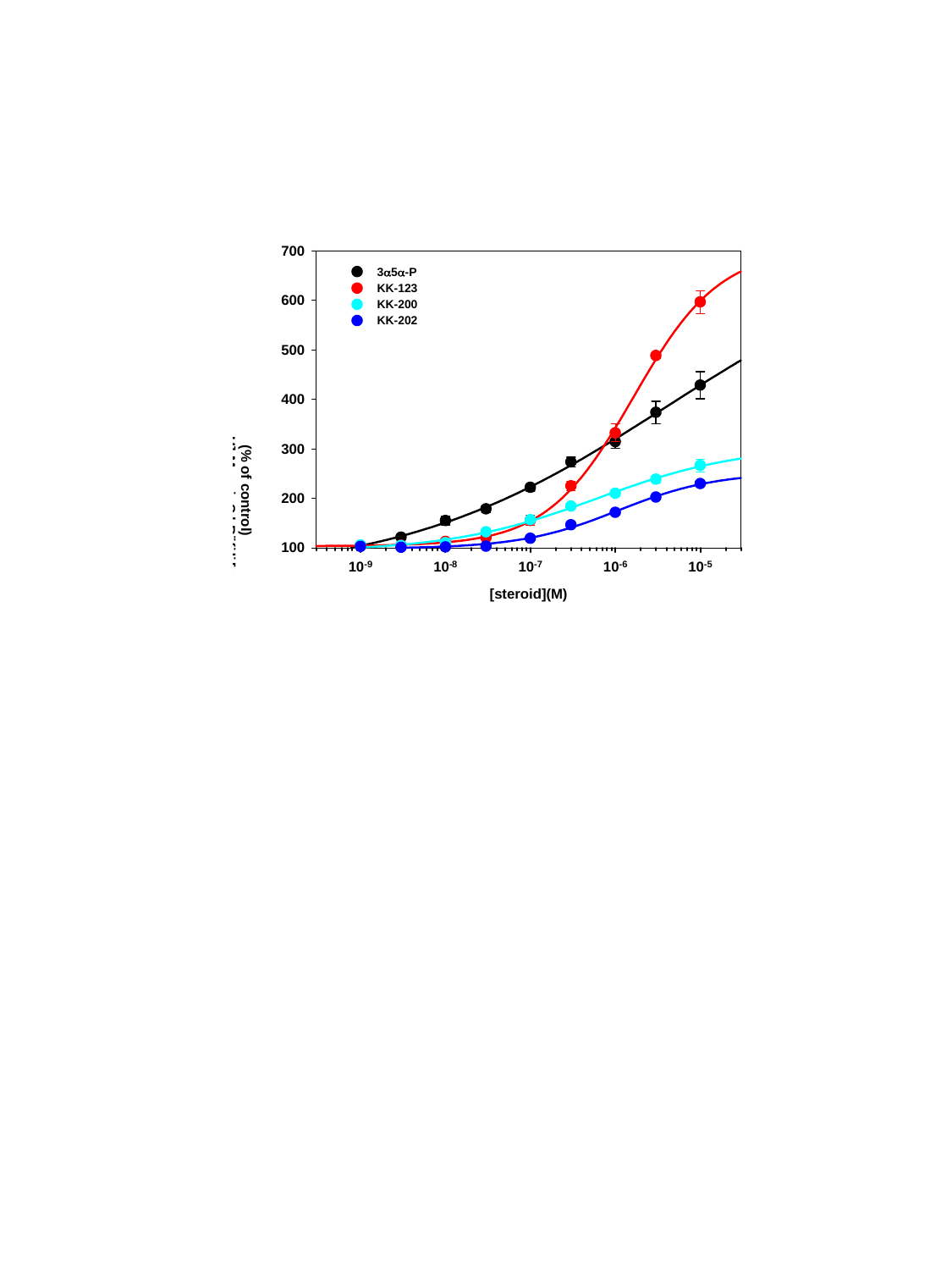

Supplement: S1 Fig — The EC50 values (in μM) are 3.9 ± 5.7 (n = 9) for allopregnanolone; 1.6 ± 0.2 (n = 9) for KK123; 0.54 ± 0.18 (n = 9) for KK200; and 1.1 ± 0.27 (n = 9) for KK202. The numerical data are included in S5 Data. EC50, half maximal effective concentration. (PPTX) [file pbio.3000157.s001.pptx]

## Slide 1
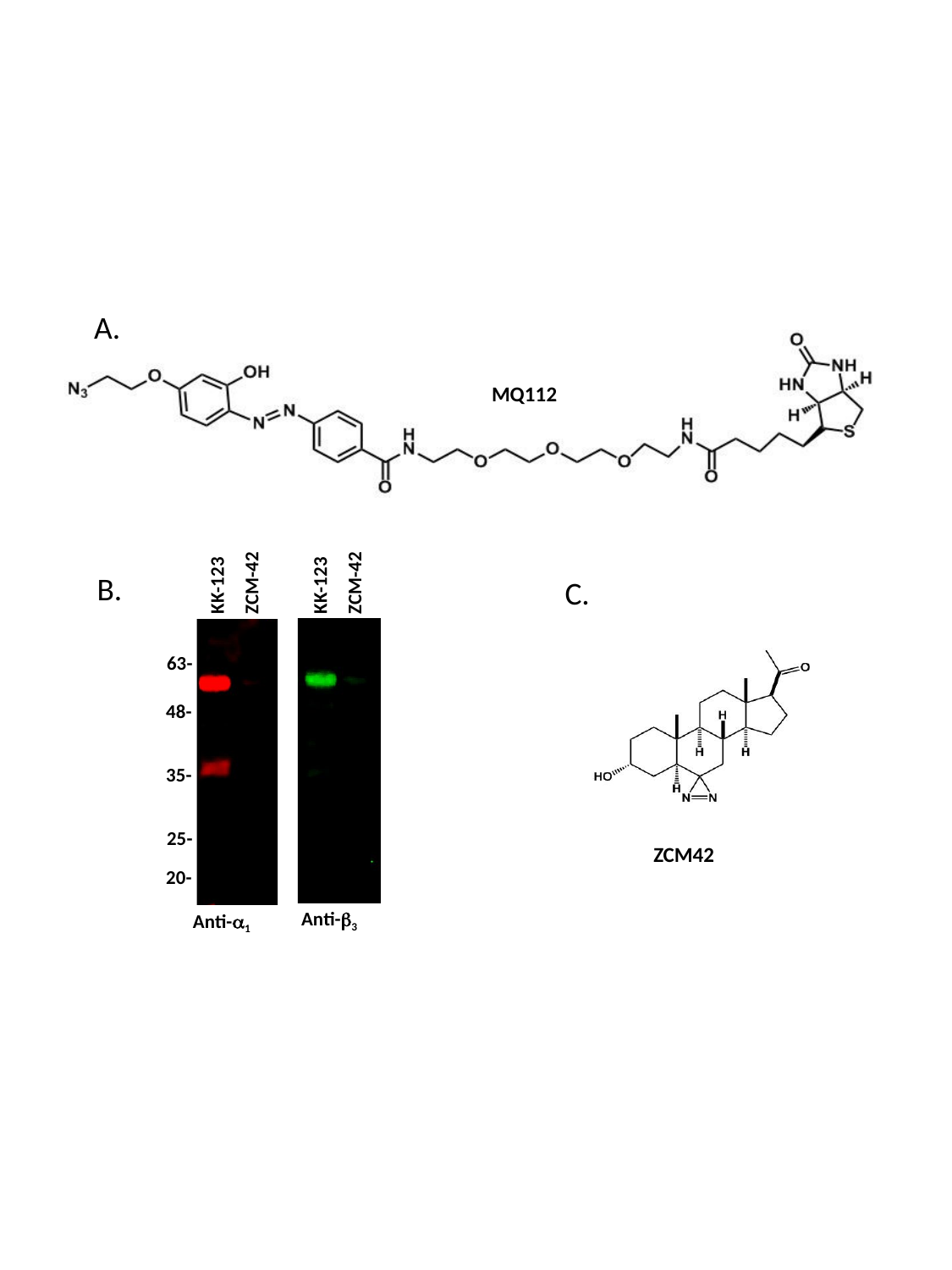

A.
MQ112
ZCM-42
ZCM-42
KK-123
KK-123
B.
C.
63-
48-
35-
25-
ZCM42
20-
Anti-b3
Anti-a1

Supplement: S2 Fig — (a) The structure of MQ112. (b) Purification of KK123 photolabeled GABAA receptor α1 and β3 subunit by MQ112, via a click reaction, visualized by western blot with anti-α1 and anti-β3. (c) The structure of ZCM42. (PPTX) [file pbio.3000157.s002.pptx]

## Slide 1
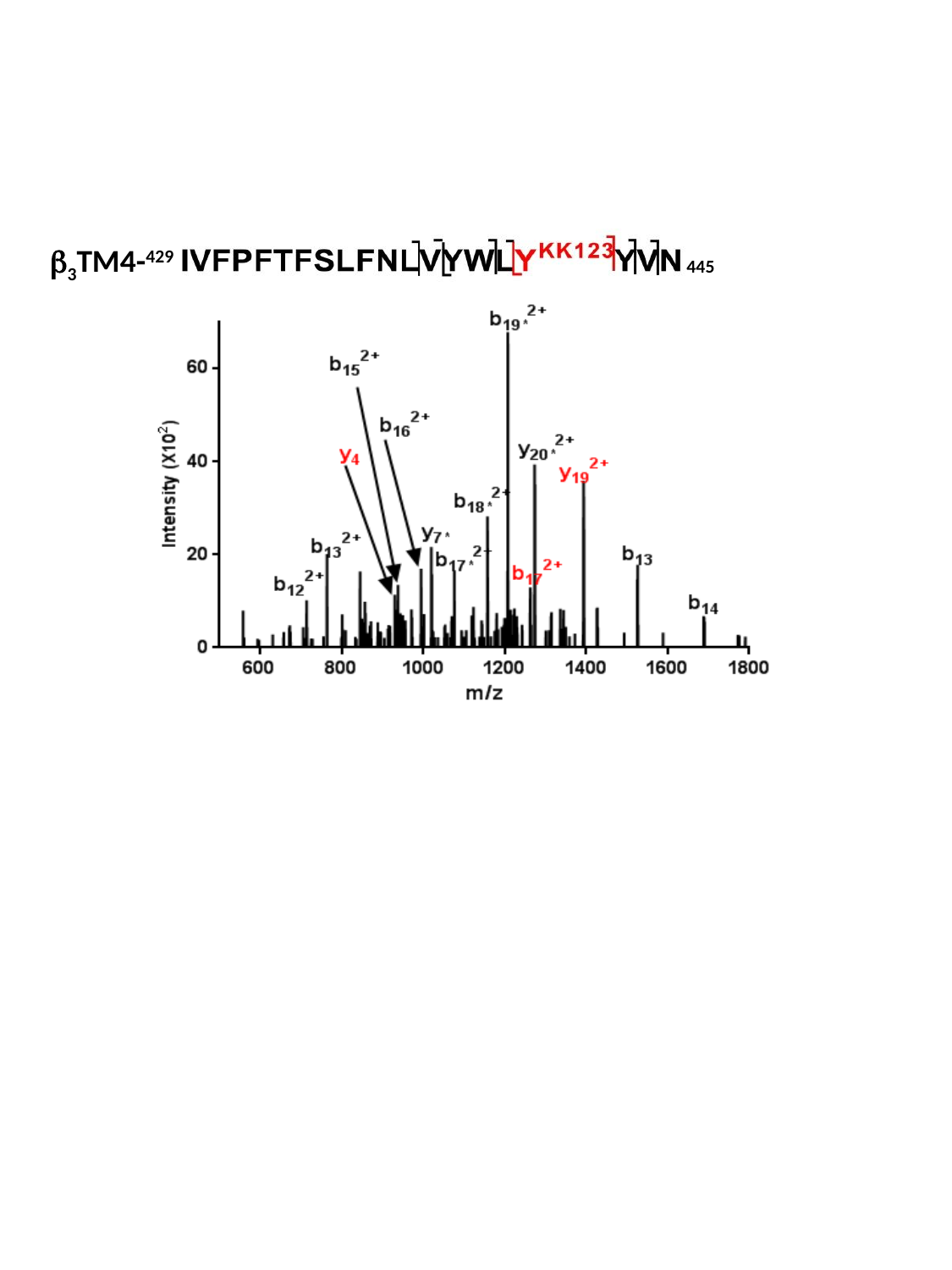

b3TM4-429
445

Supplement: S4 Fig — Y4 and b17 (in red) fragment ions containing a KK123 adduct indicate that Y442 is photolabeled by KK123. The fragment ions with neutral loss of the adduct are labeled as b17*2+, b18*2+, b19*2+, and y20*2+. The numerical data are included in S6 Data. (PPTX) [file pbio.3000157.s004.pptx]
