# Supplementary material for: Multiple functional neurosteroid binding sites on GABAA receptors
Source: PLoS Biol. 2019 Mar 7;17(3):e3000157. doi: 10.1371/journal.pbio.3000157 (PMC6424464; doi:10.1371/journal.pbio.3000157)
Supplement: S3 Fig — (PPTX) [file pbio.3000157.s003.pptx]

## Slide 1
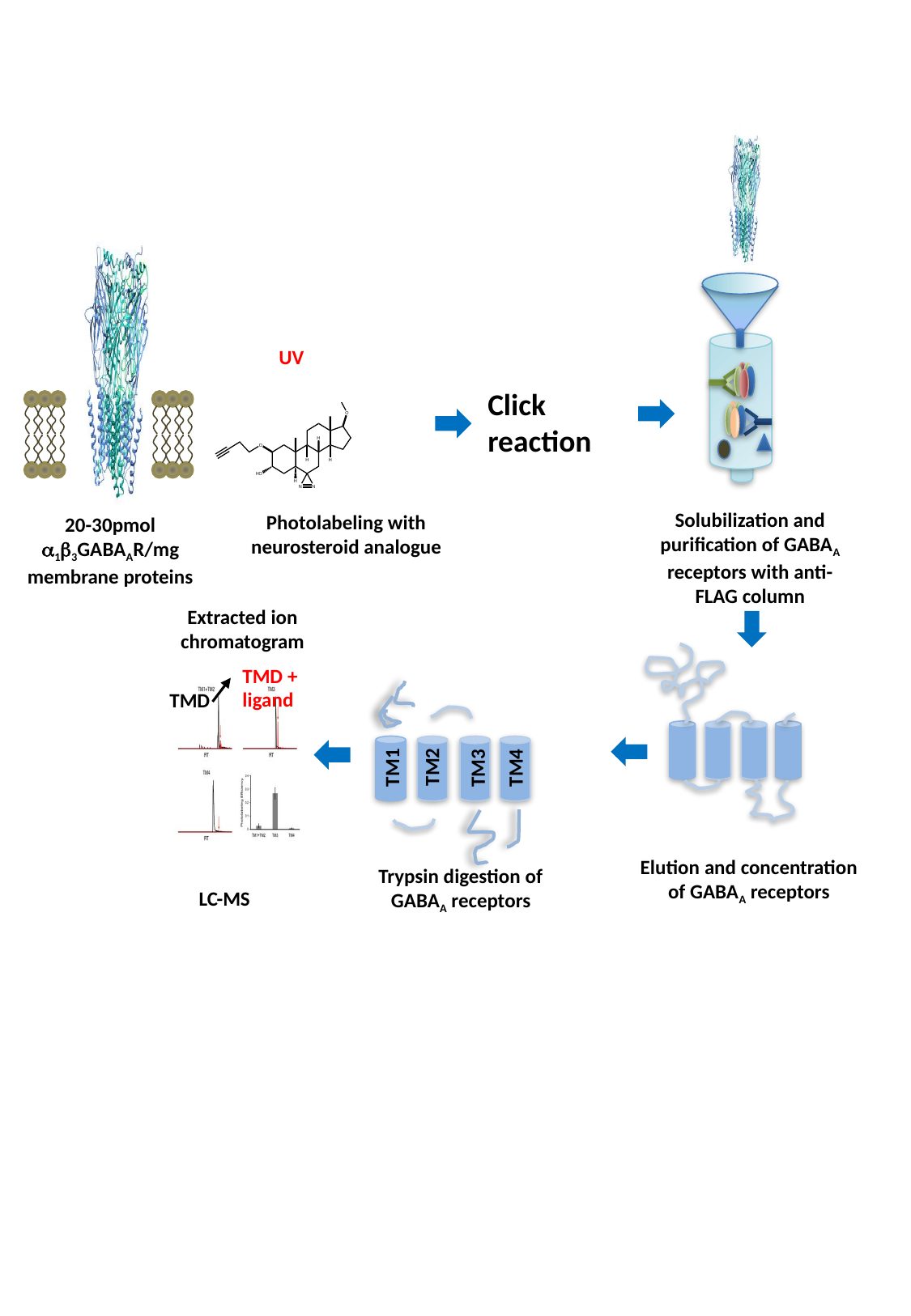

20-30pmol a1b3GABAAR/mg membrane proteins
UV
Click reaction
Solubilization and purification of GABAA receptors with anti-FLAG column
Photolabeling with neurosteroid analogue
Extracted ion
chromatogram
TMD + ligand
TM2
TM1
TM4
TM3
TMD
Elution and concentration of GABAA receptors
Trypsin digestion of GABAA receptors
LC-MS
