# Supplementary material for: Multiple functional neurosteroid binding sites on GABAA receptors
Source: PLoS Biol. 2019 Mar 7;17(3):e3000157. doi: 10.1371/journal.pbio.3000157 (PMC6424464; doi:10.1371/journal.pbio.3000157)
Supplement: S5 Fig — Each panel shows the structural model of an α1β3 GABAA receptor (based on the structure of the β3 homomeric GABAA receptor [PDB 4COF] superimposed on models based on the X-ray crystallographic structures of the α5-β3 chimera (panels a and c) or the α1-GLIC chimera (panels b and d). Docking of allopregnanolone to β3-α1 intersubunit site is shown above (panels a and b) and to the α1 intrasubunit site below (panels c and d). The β3 subunit in the α1β3 GABAA receptor model is colored light gray, and the α1 subunit is colored dark gray. TMDs in the α5-β3 chimera (panels a and c) are colored gold; TMDs in the α1-Glic chimera (panels b and d) are pink. The preferred pose of allopregnanolone docking to the α1β3-GABAA receptors is shown in cyan and to the chimeric receptors in dark red. The KK123 photolabeled residue α1-Y415 is colored in red, and the KK200 photolabeled residue α1-N408 is colored green. The canonical neurosteroid binding residues Q242, F301, and the new mutation residue—V227—are colored yellow. GLIC, Gloeobacter ligand-gated ion channel; TMD, transmembrane domain. (PPTX) [file pbio.3000157.s005.pptx]

## Slide 1
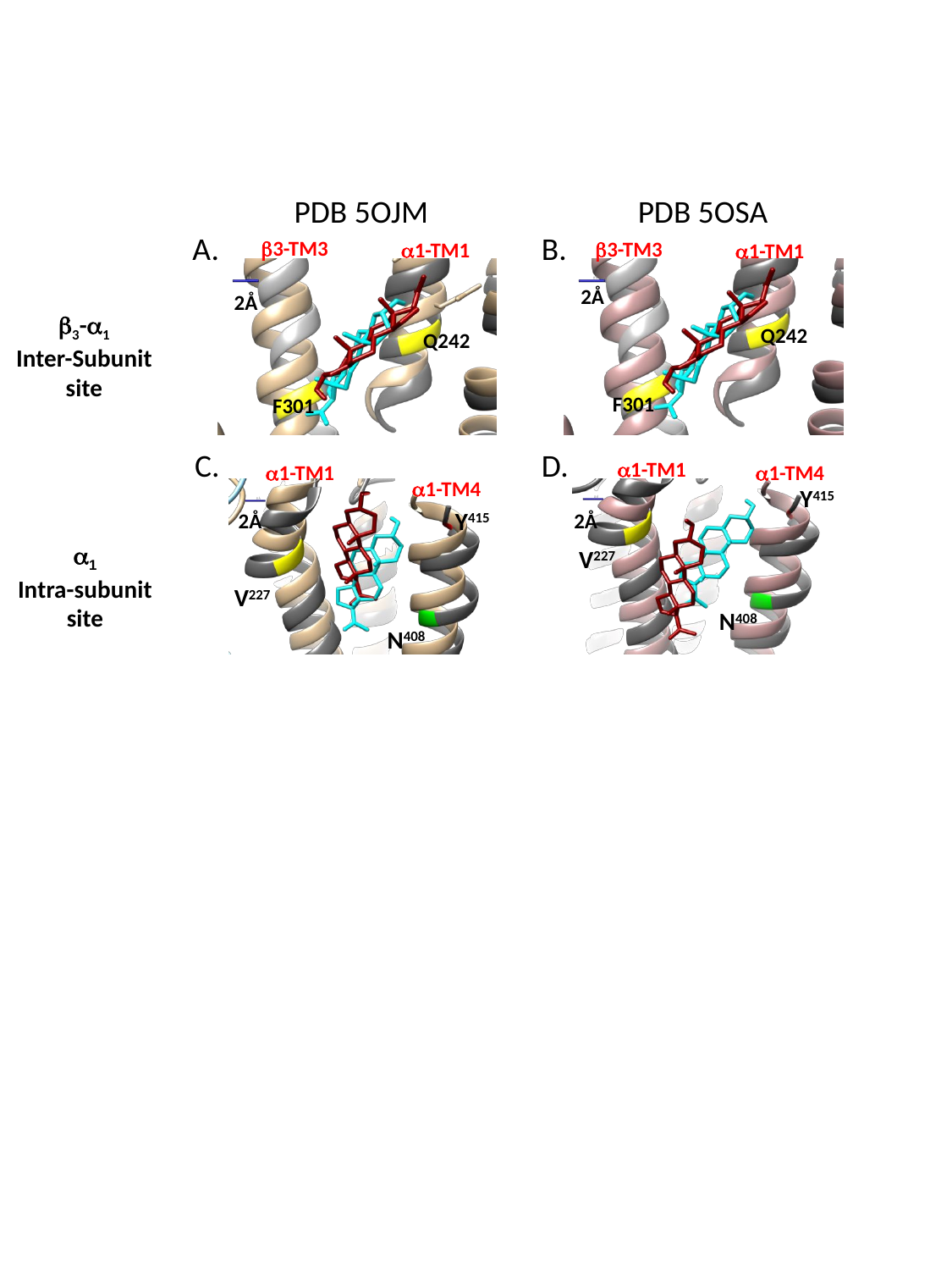

PDB 5OJM
A.
b3-TM3
a1-TM1
2Å
Q242
F301
PDB 5OSA
B.
b3-TM3
a1-TM1
2Å
Q242
F301
b3-a1
Inter-Subunit
site
C.
a1-TM1
a1-TM4
Y415
2Å
V227
N408
D.
a1-TM1
a1-TM4
Y415
2Å
V227
N408
a1
Intra-subunit
site
