# Supplementary material for: Multiple functional neurosteroid binding sites on GABAA receptors
Source: PLoS Biol. 2019 Mar 7;17(3):e3000157. doi: 10.1371/journal.pbio.3000157 (PMC6424464; doi:10.1371/journal.pbio.3000157)
Supplement: S1 Table — Data are presented as area under the curve of selected ion chromatograms of photolabeled peptides as a percentage of the area under the curve of corresponding nonphotolabeled peptides. (DOCX) [file pbio.3000157.s006.docx]

**Supplemental table 1**

|  | **KK123 (%)** | **KK200 (%)** | **KK202 (%)** |
| --- | --- | --- | --- |
| **α_1_-TM4 intrasubunit site** | **0.38±0.04** | **0.06±0.08** | **0** |
| **β_3_-TM4 intrasubunit site** | **3.19±2.26** | **0** | **0.53±0.63** |
| **β_3_- α_1_ intersubunit site** | **0** | **1.54±0.03** | **0.42±0.23** |
