# Supplementary material for: Multiple functional neurosteroid binding sites on GABAA receptors
Source: PLoS Biol. 2019 Mar 7;17(3):e3000157. doi: 10.1371/journal.pbio.3000157 (PMC6424464; doi:10.1371/journal.pbio.3000157)
Supplement: S2 Table — Potentiation is expressed as potentiation response ratio, calculated as the ratio of the peak responses in the presence of GABA and neurosteroids to the peak response in the presence of GABA alone. The GABA concentrations were selected to generate a response of 5%–15% of the response to saturating GABA. Data are shown as mean ± SD (number of cells). One-way ANOVA followed by Dunnett’s multiple comparison to the control wild-type group was used for statistical analysis. *p < 0.05; **p < 0.01; and ***p < 0.001. (DOCX) [file pbio.3000157.s007.docx]

**Supplemental table 2.**

| **Receptor** | **Potentiation by**  **0.1 μM 3α5αP** | **Potentiation by**  **1 μM 3α5αP** | **Potentiation by 0.1 μM 3α5βP** | **Potentiation by**  **1 μM 3α5βP** |
| --- | --- | --- | --- | --- |
| α_1_β_3_ wild-type | 4.8±1.4 (6) | 8.4±3.4 (6) | 3.7±1.7 (6) | 7.7±4.3 (6) |
|  |  |  |  |  |
| α_1_^F407A^β_3_ | 5.3±2.1 (6) | 9.0±4.4 (6) | 3.0±0.9 (5) | 5.1±1.6 (5) |
| α_1_^N408A/Y411F^β_3_ | 1.4±0.2 (6) * | 3.5±1.6 (10) ** | - | - |
| α_1_^W412A^β_3_ | 3.2±0.7 (5) | 6.3±2.2 (5) | 4.3±1.1 (6) | 7.1±2.7 (6) |
| α_1_^W412L^β_3_ | 5.2±1.4 (5) | 9.5±3.1 (5) | 5.2±1.6 (5) | 10.8±5.8 (5) |
| α_1_^Y415A^β_3_ | 4.9±1.7 (5) | 9.7±4.2 (5) | 3.9±1.4 (5) | 6.5±2.3 (5) |
| α_1_^F289A^β_3_ | 2.8±0.5 (6) | 4.9±1.0 (6) | 3.0±0.7 (4) | 5.5±1.3 (4) |
| α_1_^V227W^β_3_ | 2.0±0.6 (5)* | 2.9±1.0 (5)** | 2.2±0.7 (6) | 3.1±0.9 (6) |
|  |  |  |  |  |
| α_1_β_3_^F438A^ | 3.3±0.7 (5) | 5.7±2.1 (5) | 4.6±1.3 (5) | 11.9±6.9 (5) |
| α_1_β_3_^W443A^ | 4.9±1.4 (4) | 9.9±3.3 (4)* | 3.5±0.6 (5) | 7.8±3.0 (5) |
| α_1_β_3_^W443L^ | 4.5±1.6 (5) | 13.9±2.6 (5) | 5.5±2.2 (5) | 12.6±4.9 (5) |
| α_1_β_3_^Y445A^ | 3.9±2.1 (10) | 11.7±4.7 (10) | 2.5±0.7 (9) | 6.8±4.3 (9) |
| α_1_β_3_^Y284F^ | 4.1±2.5 (10) | 10.3±2.9 (10) | 2.3±1.0 (5) | 7.2±1.9 (5) |
| α_1_β_3_^I222W^ | 4.0±0.6 (5) | 7.0±1.7 (5) | 3.2±0.5 (4) | 5.3±1.7 (4) |
