# Supplementary material for: Multiple functional neurosteroid binding sites on GABAA receptors
Source: PLoS Biol. 2019 Mar 7;17(3):e3000157. doi: 10.1371/journal.pbio.3000157 (PMC6424464; doi:10.1371/journal.pbio.3000157)
Supplement: S3 Table — Direct activation is expressed in units of open probability. Data are shown as mean ± SD (number of cells). One-way ANOVA followed by Dunnett’s multiple comparison to the control wild-type group was used for statistical analysis. *p < 0.05 and ***p < 0.001. (DOCX) [file pbio.3000157.s008.docx]

**Supplemental table 3.**

| **Receptor** | **Direct activation by 10 μM 3α5αP** | **Direct activation by 10 μM 3α5βP** |
| --- | --- | --- |
| α_1_β_3_ wild-type | 0.034±0.012 (5) | 0.110±0.052 (4) |
|  |  |  |
| α_1_^F407A^β_3_ | 0.073±0.035 (5) | 0.036±0.020 (5) |
| α_1_^N408A/Y411F^β_3_ | 0.019±0.0078 (5) | - |
| α_1_^W412A^β_3_ | 0.081±0.042 (5) | 0.070±0.027 (5) |
| α_1_^W412L^β_3_ | 0.18±0.10 (8) | 0.41±0.17 (5)*** |
| α_1_^Y415A^β_3_ | 0.44±0.13 (7)*** | 0.28±0.09 (10)* |
| α_1_^F289A^β_3_ | 0.089±0.014 (4) | 0.057±0.011 (4) |
| α_1_^V227W^β_3_ | 0.012±0.017 (9)* | 0.016±0.009 (5)* |
|  |  |  |
| α_1_β_3_^F438A^ | 0.095±0.123 (8) | 0.089±0.049 (5) |
| α_1_β_3_^W443A^ | 0.10±0.05 (5) | 0.071±0.031 (5) |
| α_1_β_3_^W443L^ | 0.017±0.011 (5) | 0.018±0.015 (5) |
| α_1_β_3_^Y445A^ | 0.75±0.26 (7)*** | 0.66±0.10 (5)*** |
| α_1_β_3_^Y284F^ | 0.11±0.07 (6) | 0.09±0.05 (4) |
| α_1_β_3_^I222W^ | 0.23±0.26 (9) | 0.25±0.18 (10) |
|  |  |  |
|  |  |  |
