# Supplementary material for: Multiple functional neurosteroid binding sites on GABAA receptors
Source: PLoS Biol. 2019 Mar 7;17(3):e3000157. doi: 10.1371/journal.pbio.3000157 (PMC6424464; doi:10.1371/journal.pbio.3000157)
Supplement: S4 Table — Free energies of binding are predicted by Vina and are in kilocalories per mole. (DOCX) [file pbio.3000157.s009.docx]

Supplemental table 4.

| **Receptor** | **α_1_ intrasubunit site affinity** | **β_3_ intrasubunit site affinity** | **β_3_-α_1_ intersubunit site affinity** |
| --- | --- | --- | --- |
| **α_1_β_3_ model** | -6.6 | -5.4 | -7.8 |
| **50JM** | -6.8 |  | -8.9 |
| **50SA** | -6.6 |  | -9.9 |
