# Supplementary material for: Multiple functional neurosteroid binding sites on GABAA receptors
Source: PLoS Biol. 2019 Mar 7;17(3):e3000157. doi: 10.1371/journal.pbio.3000157 (PMC6424464; doi:10.1371/journal.pbio.3000157)
Supplement: S1 Text — (DOCX) [file pbio.3000157.s016.docx]

**Chemical syntheses.** Solvents were either used as purchased or dried and purified by standard methodology. Extraction solvents were dried with anhydrous Na_2_SO_4_ and after filtration, removed on a rotary evaporator. Flash column chromatography was performed using silica gel (32–63 μm) purchased from Scientific Adsorbents (Atlanta, GA). NMR spectra were recorded in CDCl_3_ (unless stated otherwise) at ambient temperature at 400 MHz (^1^H) or 100 MHz (^13^C).

***Synthesis of 3-​[4-​(iodomethyl)​phenyl]​-​3-​(trifluoromethyl)​-3*H*-​Diazirine (8).***

**1-​Bromo-​4-​[(methoxymethoxy)​methyl]​-benzene (2)**

Chloromethyl methyl ether (3.06 ml, 40.35 mmol) was added to a stirred, cold solution of 4-bromobenzyl alcohol (**1**, 5.0 g, 26.9 mmol) and Hunig’s base (14.05 ml, 80.7 mmol) in CH_2_Cl_2_ (30 ml) and the reaction was stirred at room temperature for 12 h. Aqueous NaHCO_3_ (100 ml) was added and the product was extracted into CH_2_Cl_2_ (3 x 75 ml). The combined extracts were dried, filtered and the solvents removed to give an oil which was purified by flash column chromatography (silica gel eluted with 5-10% EtOAc in hexanes) to give compound **2** as a colorless liquid (6.0 g, 97%) which had: ^1^H NMR δ 7.39, (d, 2H, *J* = 8.2 Hz), 7.16 (d, 2H, *J* = 8.2 Hz), 4.61 (s, 2H), 4.46 (s, 2H), 3.32 (s, 3H); ^13^C NMR δ 136.91, 131.49, 129.43, 121.52, 95.70, 68.36, 55.38.

**1-​[4-​[(Methoxymethoxy)​methyl]​phenyl]-2,2,2-trifluoro​-ethanone (3)**

Compound **2** (6.0 g, 26 mmol) in THF was added to a stirred hot suspension of Mg turnings (947 mg, 39 mmol) in THF (100 ml) and the mixture was heated to reflux for 15 min. Ethyl bromide (0.15 ml, 2 mmol) was added to the refluxing solution to activate the magnesium and the mixture was refluxed for another 90 min. The resulting Grignard reagent in THF was cooled to room temperature and transferred using a cannula into a flask containing a cold solution of 1-​trifluoroacetyl piperidine (18.1 g, 100 mmol) in THF (15 ml). The reaction was allowed to stir for another 13 h at room temperature. Saturated aqueous NH_4_Cl was added and the product was extracted thrice into EtOAc. The combined extracts were dried, filtered and the solvents removed to give a pale yellow oil which was purified by flash column chromatography (silica gel eluted initially hexanes and then with 2-8% EtOAc in hexanes) to give compound **3** (4 g, 62%) as a liquid which had: ^1^H NMR δ 8.07 (d, 2H, *J* = 7.8 Hz), 7.54 (d, 2H, *J* = 7.8 Hz), 4.75 (s, 2H), 4.70 (s, 2H), 3.42 (s, 3H).

**1-​[4-​[(Methoxymethoxy)​methyl]​phenyl]​-​2,​2,​2-​trifluoro-​ethanone oxime (4)**

Compound **3** (3.8 g, 15 mmol), hydroxylamine hydrochloride (6.95 g, 100 mmol) and NaOAc (16.4 g, 200 mmol) in MeOH (200 ml) were refluxed for 48 h. The reaction was cooled and the MeOH was removed. Water was added and the product was extracted trice into CH_2_Cl_2_. The combined extracts were dried, filtered and the solvents removed to give an *E/Z* mixture of compound **4** as an oil (3.8 g, 96%) which had: ^1^H NMR (*E/Z* mixture) δ 9.28 & 9.05 (b s, 1H), 7.20–7.50 (m, 4H), 4.76 & 4.74 (s, 2H), 4.66 & 4.65 (s, 2H), 3.43(s, 3H).

**1-​[4-​[(Methoxymethoxy)​methyl]​phenyl]​-2,​2,​2-​trifluoro-​​ethanone​,O-​[(4-​methylphenyl)​sulfonyl] oxime (5)**

Tosyl chloride (3.29 g 17.3 mmol) was added to a stirred, cold solution of compound **4** (3.8 g, 14.4 mmol) and triethyl amine (4.2 ml, 30 mmol) in CH_2_Cl_2_ and the reaction was stirred at 0 °C for 2 h. Aqueous saturated NaHCO_3_ was added and the product was extracted thrice into CH_2_Cl_2_. The combined extracts were dried, filtered and the solvents removed to give a residue which was then converted to compound **6** without purification. Compound **5** had: ^1^H NMR (*E/Z* mixture) δ 7.90 (m, 2H), 7.30–7.55 (m, 6H), 4.75 & 4.72 (s, 2H), 4.65 & 4.64 (s, 2H), 3.43 & 3.42 (s, 3H), 2.49 & 2.47 (s, 3H).

**3-​[4-​[(Methoxymethoxy)​methyl]​phenyl]​-​3-​(trifluoromethyl)​-3*H*-diazirine (6)**

Freshly condensed anhydrous ammonia (20 mL) was added to a stirred, cold (–78 °C) solution of compound **5** (6.0 g, 14.4 mmol) in CH_2_Cl_2_ (100 ml) and the reaction was slowly warmed to room temperature and stirred for 16 h. Water was added and the product was extracted into CH_2_Cl_2_ (3 x 80 ml). The combined extracts were dried, filtered and the solvent removed to give the crude diaziridine product which was dissolved in MeOH (50 ml). Triethyl amine (10 ml) was added and then MeOH saturated with I_2_ was added until the I_2_ color persisted. 5% Aqueous sodium thiosulfate (50 ml) and then water (100 ml) were added. The product was extracted into CH_2_Cl_2_ (3 x 75 ml). The combined extracts were dried, filtered and the solvents removed to give an oil which was purified by flash column chromatography (silica gel eluted with 2-10% EtOAc in hexanes) to give compound **6** (2.6 g, 70%) as a liquid which had: ^1^H NMR δ 7.40 (d, 2H, *J* = 8.2 Hz), 7.19 (d, 2H, *J* = 8.2 Hz), 4.71 (s, 2H), 4.61 (s, 2H), 3.41(s, 3H).

**4-​[3-​(Trifluoromethyl)​-​3H-​diazirin-​3-​yl]​-benzenemethanol (7)**

Compound **6** (2.6 g, 10 mmol) in MeOH (10 ml) was added to 5-7 % dry HCl in MeOH (20 ml) and the reaction was stirred at room temperature for 13 h. Aqueous saturated NaHCO_3_ (100 ml) was added and the product was extracted into CH_2_Cl_2_ (3 x 75 ml). The combined extracts were dried, filtered and the solvent removed to give an oil which was purified by flash column chromatography (silica gel eluted with 5-20% EtOAc in hexanes) to give compound **7** (2.1 g, 97%) which had: ^1^H NMR δ 7.39 (d, 2H, *J* = 7.8 Hz), 7.20 (d, 2H, *J* = 7.8 Hz), 4.71 (s, 2H); ^13^C NMR δ 142.42, 128.15, 126.94, 126.45, 122.08 (q, *J* = 275 Hz), 63.90, 28.25 (q, *J* = 40.4 Hz).

**3-​[4-​(Iodomethyl)​phenyl]​-​3-​(trifluoromethyl)​-3*H*-diazirine (8)**

Imidazole (0.82 g, 12 mmol), triphenylphosphine (1.83g, 7 mmol) and I_2_ (2.03g, 8 mmol) in CH_2_Cl_2_ (20 ml) were stirred at room temperature for 15 min. Compound **7** (864 mg, 4 mmol) in CH_2_Cl_2_ (10 ml) was added and the reaction was stirred for 90 min at room temperature. Hexanes (100 ml) were added and the biphasic solution was stirred for 10 min. The supernatant hexanes, which were brownish in color, were added to a to a column of flash column silica gel and eluted with 2-10% EtOAc in hexanes to give compound **8** (1.1 g, 85%) as a viscous liquid which on standing crystallized to give a pale yellow solid. Compound **8** had: ^1^H NMR δ 7.40 (d, 2H, *J* = 7.8 Hz), 7.12 (d, 2H, *J* = 7.8 Hz), 4.43 (s, 2H); ^13^C NMR δ 141.07, 129.11 (2 x C), 128.65, 126.88 (2 x C), 121.99 (q, *J* = 275 Hz), 28.31 (q, *J* = 40.5 Hz).

***Synthesis of KK-200 (12).***

**Steroid 10**

Steroid **9** (301 mg, 0.9 mmol) and NaH (60% suspension in mineral oil, 0.8 g, 20 mmol) in DMF (10 ml) and THF (7 ml) were stirred at room temperature for 30 min. Compound **8** (652 mg, 2 mmol) was added and the reaction was stirred for 14 h. The reaction was cooled and 2-propanol was carefully added followed by the addition of cold water. When no NaH remained, ice followed by water (100 ml) was added and the product was extracted into EtOAc (4 x ml). The combined extracts were washed with brine, dried, filtered and the solvents removed to give an oil which was purified by flash column chromatography (silica gel eluted with hexanes followed by 2-20% EtOAc in hexanes) to give steroid **10** as an oil (205 mg, 43%) which had: ^1^H NMR: δ 7.36 (d, 2H, *J* = 8.2 Hz), 7.15 (d, 2H, *J* = 8.2 Hz), 4.53 (s, 2H), 3.92 (s, 4H), 3.38 (t, 1H, *J* = 8.2 Hz), 2.05–0.60 (m), 0.82 (s, 3H), 0.81 (s, 3H); ^13^C NMR δ 141.27, 127.83, 127.37 (2 x C), 126.34 (2 x C), 122.10 (q, *J* = 275 Hz), 109.28, 88.68, 70.72, 64.07, 54.11, 51.13, 43.64, 43.12, 37.95, 37.91, 35.98, 35.49, 35.22, 31.54, 31.40, 31.08, 28.52, 28.37, 28.12, 27.84, 23.33, 22.61, 20.79, 14.07, 11.82, 11.35.

Steroid **9** has been prepared previously [70].

**Steroid 11**

Steroid **10** (197 mg, 0.37 mmol) and *p*-toluenesulfonic acid (50 mg) in acetone (25 ml) were stirred at room temperature for 16 h. Aqueous saturated NaHCO_3_ was added and the acetone was removed. Water was added and the product was extracted into EtOAc (3 x 50 ml). The combined extracts were washed with brine, dried and the solvents removed. The crude product was purified by flash column chromatography (silica gel eluted with 15-20% EtOAc in hexanes) to give steroid **11** as an oil (163 mg, 90%) which had: ^1^H NMR: δ 7.36 (d, 2H, *J* = 8.2 Hz), 7.16 (d, 2H, *J* = 8.2 Hz), 4.53 (s, 2H), 3.38 (t, 1H, *J* = 8.2 Hz), 2.45–0.60 (m), 1.01 (s, 3H), 0.83 (s, 3H); ^13^C NMR: δ 212.03, 141.19, 127.92, 127.43 (2 x C), 126.39 (2 x C), 122.12 (q, J = 275 Hz), 88.56, 70.76, 53.88, 50.95, 46.68, 44.66, 43.12, 38.53, 38.12, 37.79, 35.70, 35.16, 31.21, 28.76, 27.83, 23.37, 21.05, 11.84, 11.45.

**Steroid 12 (KK-200)**

K-selectride (1 M in THF, 1 ml, 1 mmol) was added to steroid **11** (160 mg, 0.33 mmol) dissolved in THF at –78 °C and the reaction was stirred at –78 °C for 1h. Water (a few drops) was added and the reaction was brought to 0 °C. A 1:1 mixture of 50% aqueous H_2_O_2_ (5 ml) and aqueous 4 N NaOH (5 ml) was added and the reaction was stirred at room temperature for 90 min. Water was added and the product was extracted into EtOAc (3 x 50 ml). The combined extracts were washed with brine, dried, filtered and the solvents removed to give an oil which was purified by flash column chromatography (silica gel eluted with 20-40% EtOAc in hexanes) to give steroid **12 (KK-200)** as a white solid (140 mg, 87%): ^1^H NMR: δ 7.36 (d, 2H, *J* = 8.2 Hz), 7.16 (d, 2H, *J* = 8.2 Hz), 4.53 (s, 2H), 4.04 (s, 1H), 3.38 (t, 1H, *J* = 8.2 Hz), 2.05–0.70 (m), 0.81 (s, 3H), 0.78 (s, 3H); ^13^C NMR: δ 141.28, 127.86, 127.41 (2 x C), 126.36 (2 x C), 122.12 (q, *J* = 275 Hz), 88.73, 70.75, 66.47, 54.42, 51.22, 43.12, 39.10, 37.97, 36.12, 35.83, 35.25, 32.16, 31.51, 28.96, 28.55, 28.38, 28.14, 27.84, 23.31, 20.38, 11.84, 11.15.

***Synthesis of KK-202 (17).***

**Steroid 13**

Commercially available androsterone (870 mg, 3.0 mmol), ethylene glycol (1 ml) and *p*-toluenesulfonic acid (200 mg) were refluxed for 15 h in a Dean Stark apparatus. The reaction was cooled and saturated aqueous NaHCO_3_ was added. The organic layer was washed with brine, dried, filtered and the solvents removed. The product was purified by flash column chromatography (silica gel eluted with 20-40% EtOAc in hexanes to give known steroid **13** (950 mg, 95%) as a solid which had: ^1^H NMR δ 4.02 (b s, 1H), 3.89 (m, 4H), 2.0 –0.75 (m), 0.82 (s, 3H), 0.77 (s, 3H); ^13^C NMR δ 119.45, 66.43, 65.10, 64.48, 54.02, 50.30, 45.91, 39.06, 36.08, 35.84, 35.70, 34.13, 32.15, 31.21, 30.66, 28.93, 28.38, 22.58, 20.12, 14.37, 11.12.

Steroid **13** has been prepared previously from androsterone by the same procedure [71].

**Steroid 14**

Steroid **13** (301 mg, 0.9 mmol) and NaH (60% suspension in mineral oil, 0.8 g, 20 mmol) in DMF (10 ml) and THF (7 ml) were stirred at room temperature for 30 min. Compound **8** (652 mg, 2 mmol) was added and the reaction was stirred for 14 h. The reaction was cooled and 2-propanol was carefully added followed by cold water. When no NaH remained, ice was added to the mixture followed by water (100 ml) and the product was extracted into EtOAc (4 x 60m). The combined extracts were washed with brine, dried and the solvents removed to give an oil which was purified by flash column chromatography (silica gel eluted with hexanes followed by 2-20% EtOAc in hexanes) to give steroid **14** (240 mg, 50%) as an oil which had: ^1^H NMR δ 7.37 (d, 2H, *J* = 7.0 Hz), 7.18 (d, 2H, *J* = 7.0 Hz), 4.48 (s, 2H), 3.84 (m, 4H), 3.61 (s, 1H), 2.01–0.80 (m), 0.84 (s, 3H), 0.79 (s, 3H); ^13^C NMR δ 141.40, 128.48, 127.50, 126.44, 122.14 (q, *J* = 275 Hz), 119.47, 73.6, 68.78, 65.13, 64.49, 54.08, 50.36, 45.96, 39.60, 35.94, 35.73, 34.15, 33.02, 32.78, 31.22, 30.69, 28.44, 25.63, 22.60, 22.00, 20.18, 14.38, 11.37.

**Steroid 15**

Steroid **14** (230 mg, 0.43 mmol) and *p*-toluenesulfonic acid (100 mg) in acetone were stirred at room temperature for 15 h. Aqueous saturated NaHCO_3_ was added and the acetone was removed. The residue was dissolved in EtOAc and washed with brine, dried, filtered and the solvent removed to give an oil which was purified by flash column chromatography (silica gel eluted with hexanes followed by 5-25% EtOAc in hexanes) to give steroid **15** (195 mg, 93%) as an oil which had: ^1^H NMR δ 7.36 (d, 2H, *J* = 7.0 Hz), 7.16 (d, 2H, *J* = 7.0 Hz), 4.46 (AB q, 2H, *J* = 11.0 Hz), 3.60 (s, 1H), 2.50–0.70 (m), 0.83 (s, 3H), 0.80 (s, 3H) ; ^13^C NMR δ 221.45, 141.40, 128.47, 127.53, 126.44, 122.13 (q, *J* = 275 Hz), 73.61, 68.88, 64.49, 54.35, 51.46, 47.79, 39.60, 36.05, 35.81, 35.01, 33.04, 32.69, 31.54, 30.75, 28.25, 25.53, 22.01, 21.71, 20.03, 13.77, 11.37.

**Steroid 16**

NaBH_4_ (20 mg, 0.5 mmol) was added to steroid **16** (80 mg, 0.16 mmol) dissolvend in EtOH (7 ml) and the reaction was stirred at room temperature for 4 h. Water was added and the product was extracted into EtOAc (3 x 40 ml). The combined extracts were washed with brine, dried, filtered and the solvents removed to give an oil which was purified by flash column chromatography (silica gel eluted with hexanes followed by 2-20% EtOAc in hexanes) to give steroid **16** (60 mg, 74%) as an oil which had: ^1^H NMR δ 7.39 (d, 2H, *J* = 7.0 Hz), 7.18 (d, 2H, *J* = 7.0 Hz), 4.49 (AB q, 2H, *J* = 11.0 Hz), 3.63 (m, 2H), 2.18–0.70 (m), 0.81 (s, 3H), 0.74 (s, 3H); ^13^C NMR δ 141.39, 128.30, 127.54, 126.44, 122.13 (q, *J* = 275 Hz), 81.96, 73.71, 68.85, 54.38, 51.05, 42.97, 39.67, 36.73, 36.00, 35.52, 33.05, 32.78, 31.49, 30.49, 28.45, 28.16, 25.62, 23.33, 20.35, 14.10, 11.69, 11.43, 11.12.

**Steroid 17 (KK-202)**

Steroid **16** (40 mg, 0.08 mmol) and NaH (60% suspension in mineral oil, 80 mg, 2 mmol) in DMF (4 ml) were stirred at room temperature for 30 min. Propargyl bromide (80% w/v in toluene, 2 ml) was added and the reaction was stirred for 3 h during which time the reaction turned dark in color. The reaction was cooled to 0 °C and 2-propanol was carefully added followed by cold water. When no NaH remained, ice and then water (40 ml) were added and the product was extracted into EtOAc (4 x 30 ml). The combined extracts were washed with brine, dried, filtered and the solvents removed to give an oil which was purified by flash column chromatography (silica gel eluted with hexanes followed by 2-15% EtOH in hexanes to give crude steroid **17** (40 mg) as an oil which was purified in portions by preparative thin layer chromatography on three preparative plates that were twice eluted with 2% EtOAc in hexanes. Steroid **17** was visualized on the plate by brief exposure to iodine. Steroid **17 (KK-202)** was an oil (16 mg, 37%) which had: ^1^H NMR δ 7.38 (d, 2H, *J* = 7.4 Hz), 7.17 (d, 2H, *J* = 7.4 Hz), 4.48 (AB q, 2H, *J* = 11.0Hz), 4.15 (m, 2H), 3.61 (s, 1H), 3.52 (t, *J* = 8.2 Hz), 2.39 (s, 1H), 2.15–0.70 (m), 0.79 (s, 3H), 0.76 (s, 3H); ^13^C NMR δ 141.41, 127.88, 127.55, 126.45, 122.12 (q, *J* = 275 Hz), 88.31, 80.62, 73.72, 73.67, 68.86, 57.18, 54.37, 51.20, 42.91, 39.63, 35.97, 35.26, 36.00, 35.52, 33.05, 32.77, 31.47, 28.45, 27.63, 25.62, 23.31, 20.39, 11.73, 11.42.

***Synthesis of MQ-112 (22).***

**Compound 19**

Resorcinol (6 g, 54.4 mmol) and 2-azidoethyl 4-methylbenzenesulfonate (4.34 g, 18 mmol) were dissolved in EtOH (40 ml). KOH (1.14 g, 20.2 mmol, dissolved in 3.2 ml water) was slowly added and the reaction was heated to reflux for 3 h. After cooling to room temperature, the reaction was poured into water and the product extracted into Et_2_O (2 x 100 ml). The combined extracts were dried, filtered and the solvents removed to leave a residue which was purified by flash column chromatography (silica gel eluted with 10% EtOAc in hexanes) to give compound **19** (2.65 g, 82%) which had: ^1^H NMR δ 7.13–7.10 (m, 1H), 6.48–6.42 (m, 3H), 5.92 (s, br, 1H), 4.09 (t, *J* = 9.0 Hz, 2H), 3.55 (t, *J* = 9.0 Hz, 2H); ^13^C NMR δ 159.4, 156.8, 130.2, 108.5, 106.8, 102.2, 66.8, 50.0.

**Compound 20**

NaNO_2_ (840 mg, 12 mmol) in H_2_O (24 ml) was added dropwise at 0 °C to a suspension of methyl 4-aminobenzoate (910 mg, 6 mmol) in 6 N HCl (24 ml). After 30 min, the clear solution was added dropwise to a solution of compound **19** (1.1 g, 6 mmol) in 2:1 H_2_O:THF (90 ml) containing K_2_CO_3_ (27 g). After addition was complete, 6 N HCl was added at 0 ºC and the product was extracted into EtOAc (150 ml). The EtOAc was dried, filtered and the solvent removed to leave a residue which was purified by flash column chromatography (silica gel eluted with 10% EtOAc in hexanes) to give compound **20** (270 mg, 13%) which had: ^1^H NMR δ 13.86 (s, 1H), 8.14 (d, *J* = 8.7 Hz, 2H), 7.81 (d, *J* = 8.7 Hz, 2H), 7.74 (d, *J* = 8.6 Hz, 1H), 6.62 (d, *J* = 8.6 Hz, 1H), 6.42 (s, 1H), 4.17 (t, *J* = 9.0 Hz, 2H), 3.93 (s, 3H), 3.63 (t, *J* = 9.0 Hz, 2H); ^13^C NMR δ 166.3, 163.3, 157.9, 152.3, 135.2, 133.5, 130.7 (2 x C), 121.1 (2 x C), 109.4, 101.9, 67.2, 52.2, 49.8.

**Compound 21**

LiOH (0.3 g) dissolved in water (1 ml) was added to compound **20** (45 mg, 0.132 mmol) in THF/H_2_O (10 ml/10 ml). The mixture was stirred for 16 h, acidified with 6 N HCl until pH 4–5 and the product was extracted into EtOAc (100 ml). The combined extracts were washed with water (40 ml), dried, filtered and the solvents removed. The residue was purified by flash column chromatography (silica gel eluted with 10% MeOH in CH_2_Cl_2_) to give compound **21** (42 gm, 97%) which had: ^1^H NMR δ 8.11(d, *J* = 8.2 Hz, 2H), 7.96 (d, *J* = 8.2 Hz, 2H), 7.77 (d, 8.6 Hz, 1H), 6.65–6.63 (m, 2H), 4.26 (t, *J* = 9.0 Hz, 2H), 3.63 (t, *J* = 9.0 Hz, 2H); ^13^C NMR δ 168.1, 163.6, 157.9, 153.5, 134.1, 132.0, 131.0 (2 x C), 127.7, 122.3 (2 x C), 109.0, 102.7, 67.9, 49.9.

Compound **21** has been prepared from resorcinol previously [72].

**Compound 22**

Anhydrous THF (8 ml) was added to an oven-dried flask under N_2_. Compound **21** (42 mg, 0.128 mmol), *N*-hydroxysuccinimide (49 mg, 0.33 mmol) and *N,N’*-dicyclohexylcarbodiimide (66 mg, 0.33 mmol) were added sequentially. The reaction was stirred at room temperature for 4 h. Solvent was removed under reduced pressure and the residue was dissolved partially in ice cold EtOAc. Undissolved material was removed by filtration and the filtrate was evaporated to dryness under high vacuum. The product, a NHS ester, was purified by flash column chromatography (silica gel eluted with 40% EtOAc in hexanes) and was dissolved in DMF (10 ml). Biotin-peg-NH_2_ (66 mg, 0.16 mmol) was added at room temperature. After 16 h, DMF was removed under high vacuum and the residue was purified by flash column chromatography (silica gel eluted with 5-10% MeOH in CH_2_Cl_2_) to give product (75 gm, 81%) which had: ^1^H NMR (CD_3_OD/CDCl_3_) δ 8.12 (d, *J* = 8.6 Hz, 2H), 8.01 (d, *J* = 8.2 Hz, 2H), 7.94 (d, *J* = 9.0 Hz, 1H), 6.82 (d, *J* = 2.0 Hz, 1H), 6.80 (d, *J* = 2.0 Hz, 1H), 4.61–4.59 (m, 1H), 4.43–4.36 (m, 3H), 3.80–3.74 (m, 14H), 3.67–3.64 (m, 2H), 3.50–3.46 (m, 4H), 3.30–3.25 (m, 1H), 3.05–3.00 (m, 1H), 2.86–2.82 (m, 1H), 2.33 (t, *J* = 7.4 Hz, 2H), 1.84–1.39 (m, 9H); ^13^C NMR (CD_3_OD/CDCl_3_) δ 175.5, 168.9, 164.5, 158.1, 152.9, 136.1, 135.4, 129.4 (2 x C), 122.2 (2 x C), 109.9, 102.9, 71.3 (2 x C), 70.9, 70.8, 70.4, 68.4, 62.9, 61.1, 56.6, 50.8, 41.0, 40.8, 40.0, 36.5, 29.4, 29.1, 26.4; LC-MS Calcd for [C_33_H_45_N_9_O_8_S+H^+^]: 728.3. Found: 728.4.

70. Mappus E, Renaud M, Rolland de Ravel M, Grenot C, Cuilleron CY. Synthesis and characterization by ^1^H and ^13^C nuclear magnetic resonance spectroscopy of 17 α-hexanoic derivatives of 5 α-dihydrotestosterone and testosterone. Steroids. 1992;57(3):122-34. Epub 1992/03/01. PubMed PMID: 1621267.

71. Tchedam Ngatcha B, Luu-The V, Labrie F, Poirier D. Androsterone 3α-ether-3β-substituted and androsterone 3β-substituted derivatives as inhibitors of type 3 17β-hydroxysteroid dehydrogenase: chemical synthesis and structure-activity relationship. J Med Chem. 2005;48(16):5257-68. Epub 2005/08/05. doi: 10.1021/jm058179h. PubMed PMID: 16078844.

72. Yang YY, Grammel M, Raghavan AS, Charron G, Hang HC. Comparative analysis of cleavable azobenzene-based affinity tags for bioorthogonal chemical proteomics. Chem Biol. 2010;17(11):1212-22. Epub 2010/11/26. doi: 10.1016/j.chembiol.2010.09.012. PubMed PMID: 21095571; PubMed Central PMCID: PMCPMC3103785.
